# Microbiome and plant cell transformation trigger insect gall induction in cassava

**DOI:** 10.3389/fpls.2023.1237966

**Published:** 2023-11-29

**Authors:** Omar Gätjens-Boniche, Jose Pablo Jiménez-Madrigal, Ross W. Whetten, Sandro Valenzuela-Diaz, Alvaro Alemán-Gutiérrez, Paul E. Hanson, Adrián A. Pinto-Tomás

**Affiliations:** ^1^ Laboratorio de Biología Molecular, Escuela de Ciencias Naturales y Exactas, Campus Tecnológico Local San Carlos, Instituto Tecnológico de Costa Rica, Alajuela, Costa Rica; ^2^ Department of Forestry and Environmental Resources, North Carolina State University, Raleigh, NC, United States; ^3^ Human Microbiome Research Program, Faculty of Medicine, The Helsinki University, Helsinki, Finland; ^4^ Laboratorio de Genómica y Biodiversidad, Facultad de Ciencias, Universidad del Bío-Bío, Chillán, Chile; ^5^ Escuela de Biología, Universidad de Costa Rica, San Pedro, San José, Costa Rica; ^6^ Center for Research in Microscopic Structures and Department of Biochemistry, School of Medicine, University of Costa Rica, San José, Costa Rica

**Keywords:** *Iatrophobia brasiliensis*, *Manihot esculenta*, plant galls, metagenomics, induction mechanism, genetic transformation, endophytes

## Abstract

Several specialised insects can manipulate normal plant development to induce a highly organised structure known as a gall, which represents one of the most complex interactions between insects and plants. Thus far, the mechanism for insect-induced plant galls has remained elusive. To study the induction mechanism of insect galls, we selected the gall induced by *Iatrophobia brasiliensis* (Diptera: Cecidomyiidae) in cassava (Euphorbiaceae: *Manihot esculenta* Crantz) as our model. PCR-based molecular markers and deep metagenomic sequencing data were employed to analyse the gall microbiome and to test the hypothesis that gall cells are genetically transformed by insect vectored bacteria. A shotgun sequencing discrimination approach was implemented to selectively discriminate between foreign DNA and the reference host plant genome. Several known candidate insertion sequences were identified, the most significant being DNA sequences found in bacterial genes related to the transcription regulatory factor CadR, cadmium-transporting ATPase encoded by the *cadA* gene, nitrate transport permease protein (*nrtB* gene), and arsenical pump ATPase (*arsA* gene). In addition, a DNA fragment associated with ubiquitin-like gene *E2* was identified as a potential accessory genetic element involved in gall induction mechanism. Furthermore, our results suggest that the increased quality and rapid development of gall tissue are mostly driven by microbiome enrichment and the acquisition of critical endophytes. An initial gall-like structure was experimentally obtained in *M*. *esculenta* cultured tissues through inoculation assays using a *Rhodococcus* bacterial strain that originated from the inducing insect, which we related to the gall induction process. We provide evidence that the modification of the endophytic microbiome and the genetic transformation of plant cells in *M*. *esculenta* are two essential requirements for insect-induced gall formation. Based on these findings and having observed the same potential DNA marker in galls from other plant species (ubiquitin-like gene *E2*), we speculate that bacterially mediated genetic transformation of plant cells may represent a more widespread gall induction mechanism found in nature.

## Introduction

Insect galls are abnormal structures developed by the presence and stimuli of insects in the host plant. Insect-induced plant galls are specialised plant tissues with an organised arrangement of cells and predetermined growth. The size, structure, and metabolism of galls are under the control of gall-forming insects and host plant species (Rohfritsch and Shorthouse, 1982; Leitch, 1994; Raman, 2011).

Insect gall tissues exhibit biochemical and cytological modifications that provide them with a higher nutritional quality than the surrounding plant tissue, thus facilitating a continuous source of food and additional benefits to the inducing insect (Koyama et al., 2004; Nabity et al., 2013; Ferreira et al., 2017). Most galls contain highly specialised tissue known as nutritive tissue, characterised by high concentrations of sugar (Nogueira et al., 2018), lipids, proteins, nitrogen, and a variety of other compounds (Shorthouse and Rohfritsch, 1992; Huang et al., 2015; Isaias et al., 2018).

The ability to induce galls in plants has emerged several times among and within insect orders, with representatives of gall-inducing species currently known in Diptera, Hymenoptera, Hemiptera, Coleoptera, Lepidoptera, and Thysanoptera (Mani, 1992; Espírito-Santo and Fernandes, 2007). In each order, the ability to form such structures appears to have an independent origin (Nyman and Julkunen, 2000; Ronquist et al., 2015).

In addition to insects, plant galls can be induced by mites and nematodes (Favery et al., 2016; de Lillo et al., 2018; Harris and Pitzschke, 2020; Olmo et al., 2020; Desnitskiy et al., 2023). Some fungi and bacteria species and even some viruses can also induce primary gall-like growths or, simply, neoplasm formation with low levels of cell differentiation (Ananthakrishnan, 1998; Raman, 2011; Gätjens-Boniche, 2019; Harris and Pitzschke, 2020). Examples of gall-like growths induced by microorganisms include *Agrobacterium tumefaciens* (crown gall), *Rhodococcus fascians*, *Pseudomonas savastanoi*, *Xanthomonas citri*, *Pantoea agglomerans*, *Taphrina betulina* (witches broom), and *Ustilago esculenta* (Swarup et al., 1991; Jump and Woodward, 1994; Chalupowicz et al., 2009; You et al., 2011; Dolzblasz et al., 2018; Harris and Pitzschke, 2020). Of these, *A. tumefaciens* is the best studied, as its habit of genetically transforming plant cells has found extensive use in plant biotechnology (Kavipriya et al., 2019; Song et al., 2019; Lian et al., 2022). Because *A. tumefaciens*-mediated genetic transformation of plant cells is the most understood mechanism of plant gall formation (Chou et al., 2022; Hopp et al., 2022; Azizi-Dargahlou and Pouresmaeil, 2023), this constitutes the best referenced system to propose alternative induction mechanisms in which complex gall formation caused by insects such as cynips (Hymenoptera) and cecidomyiids (Diptera) (Sinnott, 1960; Raman, 2011) is also the result of a based plant cell genetic transformation.

A large number of endosymbiotic bacteria in different insect groups, including gall-inducing insects, have been reported in several studies (Campbell et al., 2015; El-Sayed and Ibrahim, 2015; Gutzwiller et al., 2015; Michell and Nyman, 2021; Yang et al., 2021; Coolen et al., 2022; Yang et al., 2022). Insect-associated microorganisms could be important mediators of interactions between insects and plants (Hammer and Bowers, 2015; Sugio et al., 2015; Wielkopolan and Jakubowska, 2021; Coolen et al., 2022). Symbiotic relationships between inducing insects and microorganisms have been hypothesised to be involved in plant gall development (Hansen and Moran, 2014; Tooker and Helms, 2014; Gätjens-Boniche, 2019; Klimov et al., 2022). Delivery of bacteria under natural or artificial conditions by insect vectors has been reported in many insect–plant interactions (Zeidan and Czosnek, 1994; Galambos et al., 2021; Wielkopolan and Jakubowska, 2021; Ratcliffe et al., 2022). Insect-vectored bacteria in plants have been described in well-known systems such as the Huanglongbing (HLB) disease of citrus caused by the phytopathogenic bacterium Candidatus *Liberibacter asiaticus* (CLas), which is transmitted by the psyllid *Diaphorina citri.* Likewise, acquisition and effective delivery of *A. tumefaciens* by the whitefly *Bemisia tabaci* was demonstrated by Zeidan and Czosnek (1994).

It has been hypothesised that phytohormone elicitor molecules delivered by the insect inducer (Rohfritsch and Shorthouse, 1982; Tooker and Helms, 2014; Ponce et al., 2021) or indirectly by an associated microorganism (Giron et al., 2013; Bartlett and Connor, 2014; Giron et al., 2016), and effector proteins secreted by the gall-inducing insect (Zhao et al., 2015; Cambier et al., 2019; Zhao et al., 2019; Korgaonkar et al., 2021) may be the main triggering stimuli responsible for the gall induction process.

Cassava (*Manihot esculenta* Crantz) is a widely cultivated crop in Africa, Asia, and Central and South America that provides an important food source for millions of people worldwide. Cassava plants can be grown yearlong in the tropics. Moreover, it can be easily propagated in greenhouse and *in vitro* conditions. Owing to its versatility and potential, plant breeding programmes and international consortiums have invested significant resources in understanding cassava genetics. This has led to the creation of genetic linkage maps and chromosome-scale genome assembly (Wang et al., 2014; ICGMC (International Cassava Genetic Map Consortium), 2015; Lyons et al., 2022). Cassava plants are subject to gall formation, particularly cylindrical galls induced by the Cecidomyiidae, *Iatrophobia brasiliensis* (Montaldo, 1977; Rivera Hernández, 2011). However, detailed studies are lacking on the induction and formation processes of this gall.

Here, we tested the hypothesis proposed by Gätjens-Boniche (2019), who postulated that characteristic hyperplasia in the initial phase of gall induction can be triggered by the insertion of exogenous genetic elements into the genome of plant cells through an endosymbiotic bacterium originating from the inducing insect. To test this hypothesis, we employed a combination of genetic marker analyses, metagenomic analyses, and experimental gall induction in cassava plants. Taken together, our findings provide support for the role of microorganisms and genetic transformation in gall induction mechanisms.

## Materials and methods

### Galling insect model

**Figure 1:**
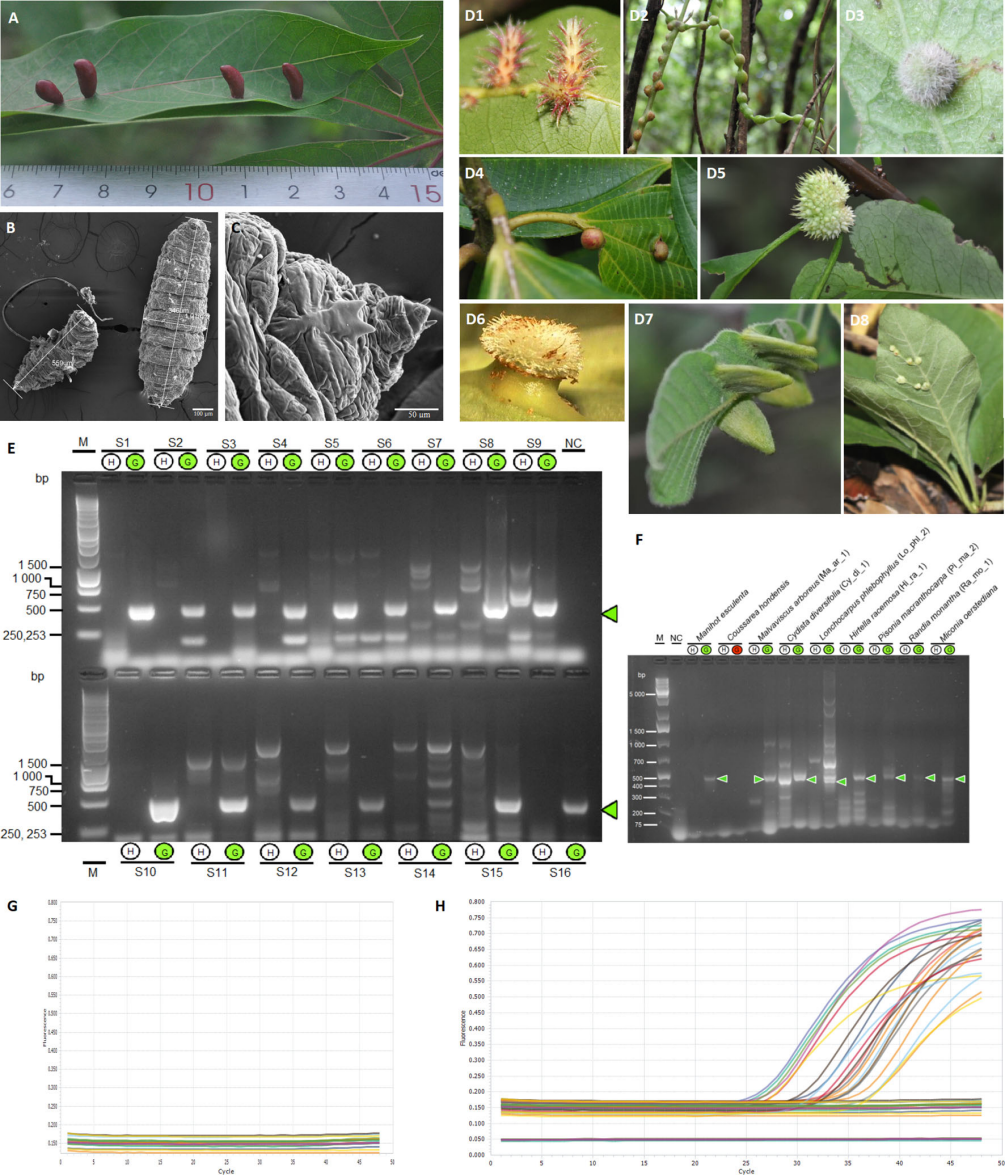
Identification of a potential universal marker in different gall systems. **(A)** Gall induced by the Cecidomyiidae *Iatrophobia brasiliensis* in *Manihot esculenta* (Montaldo, 1977). **(B)** Scanning electron micrograph of *I*. *brasiliensis* larva. **(C)** Scanning electron micrograph of the ventral area near the head of *I*. *brasiliensi*s larva. **(D1)** Gall induced in *Hirtella racemosa* Lam. (Chrysobalanaceae), morphotype Hi_ra_1, by an unidentified Cecidomyiidae. **(D2)** Gall induced in *Cydista diversifolia* (Kunth) Miers (Bignoniaceae), morphotype Cy_di_1 by an unidentified Cecidomyiidae. **(D3**) Gall induced in *Malvaviscus arboreus* Dill. ex Cav. (Malvaceae), morphotype Ma_ar_1, by an unidentified Cecidomyiidae. **(D4)** Gall induced in *Miconia oerstediana* (Melastomataceae) by an unidentified Cecidomyiidae. **(D5)** Gall in *Pisonia macranthocarpa* (Donn. Sm.) Donn. Sm. (Nyctaginaceae), morphotype Pi_ma_4, induced by an unknown insect species. **(D6)** Gall in *Coussarea hondensis* (Standl.) C.M. Taylor & W.C. Burger (Rubiaceae) induced by an unknown insect. **(D7)** Gall induced in *Lonchocarpus phlebophyllus* Standl & Steyerm. (Fabaceae), morphotype Lo_phl_1, induced by a Psyllidae. **(D8)** Gall induced in *Randia monantha* Benth. (Rubiaceae), morphotype Ra_mo_1, induced by an unknown insect species. **(D2–D8)** Gätjens-Boniche et al. (2021). **(E)** Agarose gel electrophoresis of DNA fragments (specific gall fragment marker, SGF) amplified by PCR, comparing healthy leaf tissue DNA samples **(H)** and gall tissue DNA samples **(G)**. Lane M, molecular weight marker (1 kb ladder); line NC, negative control (reagents only); lines S1–S16, samples of healthy leaf and gall tissues growing in the same plant organ (pair-compared). **(F)** Gel electrophoresis of PCR products using primers for the specific gall fragment marker (SGF) in gall morphotypes of different host plant species. Lane M, molecular weight marker (Gene Ruler 1 KB Plus); line NC, negative control (reagents only); lines 3–20, samples of healthy leaf and gall tissues of different plants growing in the same plant organ (pair-compared). The green circle indicates a positive sample for the specific gall fragment amplification **(E, F)**. **(G, H)** Real-time PCR by Taq Man Probe showing the detection of the specific gall fragment from gall DNA samples of *Manihot esculenta* (amplification plot H) and from healthy leaf samples (amplification plot G). Each trace shows the ΔRn (normalised net fluorescence signal of the PCR product) plotted against the number of PCR cycles.

We chose the cylindrical gall induced in Euphorbiaceae *M. esculenta* Crantz (cassava) (**Figure 1A**) by the Cecidomyiidae *I. brasiliensis* (Montaldo, 1977; Rivera Hernández, 2011) as our biological model (**Figure 1B**). The cassava genome has been well documented (Wang et al., 2014; ICGMC (International Cassava Genetic Map Consortium), 2015; Lyons et al., 2022), allowing the identification of potential alterations or insertions from exogenous sources.

Additionally, six prosoplasmic gall morphotypes (greater structural complexity) and two kataplasmic galls (low structural complexity) were selected to validate the general induction hypothesis (**Figure 1D**). For details, see **Supplementary Text**.

Samples were obtained from several plant specimens collected in the Guanacaste Conservation Area, Guanacaste, Costa Rica, as well as around the Santa Clara community, San Carlos, Alajuela, Costa Rica. A voucher specimen of each host plant species and galls was deposited at the Cecidiarium (specialised gall herbarium), established at the Instituto Tecnológico de Costa Rica.

### Microdissection of salivary glands from *Iatrophobia brasiliensis* larvae

Larval salivary glands from *I. brasiliensis* were extracted by microdissection in a biosafety flow-hood (High Ten, Model 3BH-24), under sterile conditions (**Figure S1**). Larvae were surface decontaminated according to the protocol established by Zahner et al. (2008) with some modifications. Consecutive rinses were done for 2 min in 3% sodium hypochlorite and then 70% ethanol, followed by a rinse in sterile nuclease-free water. All dissected salivary glands were collected and employed in the DNA extraction. All life stages were surface cleaned.

### Isolation and characterisation of endosymbiotic insect bacteria

Two colony-forming units (CFUs) were preliminarily isolated from the larval head of *I. brasiliensis* under sterile conditions. The dissected larvae were in instars one or two. The larvae were extracted live from the inner chamber of the gall, and only active larvae were collected. Different parts of the larva, such as the head and segments of the digestive system, were placed in YEB 1× culture medium (Piñol et al., 1996) to obtain a primary bacterial culture. Serial dilutions were made from the original culture to obtain single colonies (CFUs). The cells were recovered and maintained in YEB 1× solid and liquid suspension to 4–8°C and in glycerol (20% w/v) at −80°C. Each of the CFUs was named isolated symbiotic bacteria (ISB).

ISB 2 formed short rods and macroscopically coccoid-like elements and produced round, entire, convex, orange/pinkish colonies with smooth matte surfaces on YEB 1× Bacto agar medium after 4–5 days of incubation at 26°C. The bacterium showed features of a pinkish, flat, circular, slightly irregular border, variable length with curved shapes in some of them, and a mucous texture; it was a Gram-positive bacillus (**Figure S2**).

Molecular characterisation of bacteria was done through 16S sequencing using the procedures described by Rainey et al. (1996) and Hanshew et al. (2013), with some modifications. Taxonomic identification of the endosymbiotic bacteria was done using Kraken2 (Wood et al., 2019), the same for the endophytic bacteria, as described later. Details of the analytical pipeline are described below. Bacterial strains were identified by 16S gene sequencing before each inoculation assay to induce gall formation in the selected cassava tissues. We prepared templates for PCR either by total DNA extraction or by lysis of CFU bacteria growing in a solid culture medium. Bacterial cells were diluted in Elution Buffer (10 mM Tris, pH 8.0) (Qiagen, Hilden, Germany). Samples were lysed by vortexing and heating at 95°C for 5 min. The filtrate was then centrifuged at 8,000 *g* for 5 min.

### Isolation of culturable microorganisms associated with plant gall tissue

We isolated CFUs that potentially represent endophytic bacterial strains and one fungal growth from surface-sterilised internal sections of *M. esculenta* gall tissue sections. Seven were selected for high-throughput sequencing (HTS). Each of these CFUs was named an isolate of endophytic bacteria (IEB).

Surface tissue was sterilised following the methodology described for DNA purification. CFUs grown directly from gall slice explants were carefully collected by a sterile bacteriological loop in a sterile biosafety chamber. Primary cultured bacteria were diluted in liquid medium YEB 1× (Piñol et al., 1996) until CFUs were obtained on solid medium. This process was repeated two to three times. Bacteria in cultured media were grown for 3–4 days at 26°C. Subsequently, bacterial cells were recovered and maintained in YEB 1× solid medium and suspended in liquid medium YEB 1× at 4–8°C and in glycerol (20% w/v) at −80°C.

### DNA purification

#### Isolation of total genomic DNA from plant tissues

All tissues were sampled from plants morphologically identifiable as *M. esculenta*. Healthy leaf tissue from *M. esculenta* was checked in a stereoscope to avoid visible microgalls or some other type of foliar damage or disturbance. Prior to DNA extraction, the galls were dissected by carefully splitting them in half without damaging the larva present in the internal chamber, which was removed from the tissue. Healthy leaves and galls were washed with alkaline liquid soap and Triton X-100 detergent (dissolved in sterile water). The plant material was vortexed three times with 70% ethanol (v/v) and sterile water. Subsequently, the leaves and galls were surface sterilised according to the methodology suggested by Meyer and Hoy (2008) and Mueller et al. (2004), with some modifications. Plant materials were immersed for less than 30 s in sodium hypochlorite sequential solutions (3% NaOCl, 6% NaOCl, and 3% NaOCl) and rinsed with sterile water. The sterilisation process was conducted in a biosafety flow-hood (High Ten, Model 3BH-24). After cleaning and sterilisation, all samples were immediately stored at −80°C until DNA extraction.

Genomic DNA extraction was performed according to the methodology established by Dellaporta et al. (1983), with modifications and following column precipitation steps as described by the Dneasy Power Plant Pro Kit protocol (Mobio/QIAGEN, Carlsbad, CA, USA). The phenolic separation solution (PSS) in the Dneasy Power Plant Pro Kit was used during maceration to avoid DNA methylation. Total genomic DNA was extracted from both healthy leaf and gall tissues (different morphotypes). In the case of galls, DNA samples were collected from individual and pooled galls growing on the same leaf.

#### Genomic DNA isolation from salivary glands of inducing insects*, Iatrophobia brasiliensis*

Larval samples for DNA extraction were collected by dissecting galls from *M. esculenta*. Pools of 10 larvae of *I. brasiliensis* were decontaminated by being exposed to 3% NaOCl for 3–4 min, followed by successive 70% alcohol and water rinses under a biosafety flow-hood (High Ten, Model 3BH-24). Genomic DNA purification from the salivary glands of the insect larvae was performed using the Power Soil DNA Isolation Kit (Mobio/QIAGEN, Carlsbad, CA, USA) with the additional steps, mainly K proteinase (100 mg/mL) treatment and incubation at 65°C for 1 h. Furthermore, salivary gland DNA samples were extracted three times with phenol-chloroform isoamyl alcohol (PCI).

#### DNA isolation from endophyte and putative insect endosymbiotic bacteria

Total genomic DNA from endosymbiotic bacteria isolated from *I. brasiliensis* insects was extracted using a Dneasy Blood and Tissue Kit (Qiagen, Hilden, Germany). The same DNA extraction protocol was used for endophytic bacteria strains grown from surface-sterilised internal sections of *M. esculenta* gall tissue sections. Plasmid DNA from the isolated bacteria strains was carried out by alkaline lysis, as described by Li et al. (1995) with some modifications. The integrity and yield of bacterial genomic DNA and wild-type plasmids were checked by 0.8% agarose gel electrophoresis.

For all genomic DNA samples (plant, insect, and bacteria), quality was assessed by 260/280 and 260/230 ratios measured on a NanoDrop 8000 Spectrophotometer (Thermo Scientific, Wilmington, DE, USA).

### Modified RAPD methodology for discovering gall-associated molecular markers

Samples of healthy leaf and gall tissues growing in the same plant organ were compared to detect differentially amplified DNA fragments in the DNA of the gall and not present in the same healthy tissue. The theoretical approach of this methodology is shown in **Figure S3A**. Assays were made under standard RAPD methodology conditions with the commercial random primers OPC-06, OPI-04, OPA-03, OPD-18, OPD-03, OPE-06, OPA-17, and OPB-04 (Operon Technologies, Alameda, California, USA). In these analyses, a modified methodological approach was also carried out by simultaneously using a combination of two non-random primers of conserved sequences from *A. tumefaciens* genes along with a non-standard RAPD thermal profile. Primers from conserved *A. tumefaciens* genes were used because the genetic transformation of plant cells mediated by this bacterium is the best-studied genetic transformation in a plant system. Decamer primers derived from conserved sequences of the Isopentyl Transferase Gene (ipt) and from the iaaM gene (Tryptophan 2-monooxygenase) harboured in the transfer DNA of the Ti plasmid of *Agrobacterium* species generated the highest number of differentially amplified fragments in previous RAPD assays. Primers were designed from the alignment of conserved regions in different species that harbour these genes using DNA Star Lasergene 99 (Madison, Wisconsin, USA) and BioEdit version 4.8.10.1 (Hall, 1999). Nucleotide sequences for these genes were obtained from the National Center for Biotechnology Information (www.ncbi.nlm.nih.gov/pubmed/). Accessions for the iaaM genes were M91609, Z18270, X77327, U04358, and L33867, and for the ipt genes, they were X77327, X53945, X17428, M91610, and Z46375. RAPD reactions were performed on 200-µL sterile, pyrogen-safe, thin-walled plastic tubes for PCR RNase-DNase (Axygen, CA, USA), using 0.5 units of Dream Taq (Fermentas Life Sciences, Lithuania), PCR 1× (750 mM Tris-HCl [pH 8.8], 0.2 mM dNTPs, 1.5 mM MgCl_2_, 0.5 μM of each primer, and 10 ng of DNA), adjusted to a final volume of 25 μl with nuclease-free sterile water (Promega Corporation, Madison, Wisconsin, USA). The primer set used was ipt forward, 5′-CGGTGAACGA-3′ and iaaM reverse, 5′-TCCAATTTCT-3′. DNA was initially denatured for 3.5 min at 95°C, followed by 15 cycles of 95°C for 30 s, 34.5°C for 30 s, and 72°C for 2 min. This was followed by 35 cycles of 30 s denaturation at 95°C, 30 s annealing at 46°C, and 2 min elongation at 72°C, with a final elongation step of 72°C for 7 min. Reactions were carried out in a thermocycler PTC-200 DNA Engine (MJ-Research, Waltham, Massachusetts, USA). Samples of healthy and gall tissues growing in the same plant were compared in pairs. Each primer set and RAPD condition was carefully tested more than three times using 20 to 30 samples. Reactions were performed in a flow-hood (High Ten, Model 3BH-24).

RAPD products were analysed by 1%–1.5% agarose gel electrophoresis with 0.5 × TBE and 1× Gel Red at 75 V (Electrophoresis chamber and Power Pac 300 Bio-Rad, Hercules, California, USA). Amplicons were separated in a MultiNA automated system for DNA and RNA microchip analysis (MultiNA-Shimadzu, Tokyo, Japan). PCR products were cleaned using Promega Wizard SV gel and the PCR clean-up system, as per the manufacturer’s directions (Promega, Madison, WI).

The DNA gall fragments differentially amplified from the insect gall tissue of cassava by modified RAPD methodology were sequenced at the Centro de Investigaciones en Biología Celular y Molecular, Universidad de Costa Rica, San José, Costa Rica. Sequencing reactions were performed using dideoxynucleotide chain termination with the BigDye™ Terminator Kit (Applied Biosystems, USA) and 5 pmol of each ipt forward and iaaM reverse sequencing primers and analysed with an ABI Prism® 3700 Automated Sequencer (Applied Biosystems, USA). Sequenced fragments were aligned using DNA Star Lasergene 99 (Madison, Wisconsin, USA) and BioEdit version 4.8.10.1 (Hall, 1999).

### PCR amplification and sequencing for the specific gall marker

The DNA gall fragments differentially amplified from gall samples were used as templates to design a potential gall molecular marker based on our RAPD results. Primers were designed using Primer3 v.4.1.0 (Koressaar and Remm, 2007; Untergrasser et al., 2012). The primer sequences were 5′-CTT GAC ATG TTC TGG AGC GG-3′ for the forward primer (Primer_Gall-Forward) and 5′-AAC GAG CGT GGT ACT GTG AT-3′ for the reverse primer (Primer_Gall-Reverse) (Invitrogen, Carlsbad, CA, USA). The expected amplicon size was 471 bp. Primers were tested with DNA samples from both healthy and gall tissues from *M. esculenta* plants and other gall morphotypes, as well as with DNA extracted from insect salivary glands. Subsequently, the target gene was amplified in the isolated wild-type plasmids from two putative insect endosymbiotic bacteria of the genus *Rhodococcus* and *Pseudomonas* and in wild-type plasmids of all endophytic bacteria isolated from the cassava gall tissue. PCR was carried out with 1× PCR buffer (750 mM Tris-HCl [pH 8.8], 200 mM (NH_4_)_2_SO_4_, and 0.1% Tween 20), 1.5 mM MgCl_2_, 0.2 mM dNTPs, 2 U Taq Polymerase (Thermo Fisher Scientific, Wilmington, DE, USA), 0.5 μM forward and reverse primers (Invitrogen, Carlsbad, CA, USA), 10 ng of sample DNA, and H_2_O (Promega Corporation, Madison, Wisconsin, USA) for a final reaction volume of 25 µL. Amplification was performed in a thermal cycler PTC-200 DNA Engine (MJ-Research, Waltham, Massachusetts, USA) using the following cycling conditions: initial denaturation at 95°C for 3,5 min.; 40 cycles of 95°C for 30 s, 62°C for 45 s, and 72°C for 1 min; and final extension at 72°C for 5 min. The transition temperature between each step was 1°C/s. The assays were repeated multiple times for 170 galls and healthy tissue samples. Three samples with varying amounts of salivary glands were used in total, as were two bacterial plasmid samples. PCR reactions for these samples were repeated at least six times.

The PCR products were separated on a 1.5% agarose gel with 1 × TAE and 1× Gel Red at 75 V (Electrophoresis chamber and Power Pac 300 Bio-Rad, Hercules, California, USA). The expected amplicons were excised from the gel and purified using the QIAquick Gel Extraction Kit, following the manufacturer’s directions (QIAGEN, Hilden, Germany). Samples were sequenced using the Sanger method through a Macrogen service provider (Macrogen Inc., Seoul, Korea). Samples were purified and prepared for sequencing according to the methodology established by Macrogen. Sequencing of nonspecific amplified fragments for PCR was attempted; however, good quality and long base sequences could not be obtained, except for two of the samples. Sequenced fragments were aligned using DNA Star Lasergene 99 (Madison, Wisconsin, USA) and BioEdit version 4.8.10.1 (Hall, 1999).

All amplified fragments were aligned to the SGF consensus sequence using BioEdit version 4.8.10.1 (Hall, 1999) and Jalview version 2-a, 2.11.1.5 (Waterhouse et al., 2009). For salivary gland amplified fragments, an overlap of bases was frequent in several of the sequenced PCR fragments, which could show variants of the target DNA sequence in this insect tissue. A similar trend was observed in one of the colonies from endosymbiont *Rhodococcus* isolated from the inducer insect when their wild-type plasmids were analysed and linked to the DNA amplification profile, which could indicate polymorphic variants of their wild-type plasmids.

Both DNA fragments amplified from gall samples (RAPD modified technique) and the SGF were analysed using BLAST [National Center for Biotechnology Information (NCBI) (www.ncbi.nlm.nih.gov/pubmed/), Integrated Microbial Genomes & Microbiomes (IMG/M) system (https://img.jgi.doe.gov)].

### Detection of the specific gall fragment by real-time qPCR

A real-time PCR marker was designed and tested in healthy and gall tissue using a TaqMan probe with homology to the consensus of differentially amplified DNA from galls. TaqMan-based qPCR was carried out in a 20-μL reaction mixture containing Go Taq Master Mix 2X (Promega, USA) and 1μL of 20X TaqMan^®^Gene Expression Primer/Probe Mix (Applied Biosystem, CA, USA). Primers and the TaqMan probe were designed and synthesised from the specific gall fragment target with 0.5 μM of each primer (AI7ZYBP_Forward: 5′-TGTTCGCTGCACAGAGTTCT-3′ and AI7ZYBP_Reverse: 5′-GGCTTGAGTGCTTCGATTTCG-3′), 0.25 μM of the MGB probe, labelled with FAM reporter dye at the 5′ and non-fluorescence Quencher TAMRA at the 3′ end (AI7ZYBP_M_TCTGCCACCGGACCCT_NFQ), and 2.5μL of 5 ng/μL of extracted DNA. qPCR cycling conditions included initial denaturation at 95°C for 3 min and 35 cycles of denaturation at 95°C for 30 s and primer annealing and extension steps together at 60°C for 60 s. Three replicates of the negative template control consisting of nuclease-free water for molecular biology grade reactions were included in each amplification assay. Assays were performed on a LightCycler 96 Real-Time PCR System (Roche Diagnostics, Risch-Rotkreuz, Switzerland). We tested over 30 healthy tissue and gall samples and obtained an amplification average of 90% for the PCR marker or amplification signal in the case of qPCR. The assays were repeated more than six times for the same healthy leaf and gall samples.

### Bioinformatic analysis

#### Library preparation and sequencing

Short-read libraries were prepared using Illumina’s DNA TruSeq Nano Library preparation kit, following the manufacturer’s instructions. For the leaf and gall genomic DNA samples, 150-bp single-ended (SE) libraries were prepared and sequenced on the Illumina HiSeq2500 platform using the SBS sequencing kit version 4. For the bacterial isolates’ genomic DNA samples, 250-bp paired-ended (PE) libraries were prepared and sequenced in the Illumina MiSeq platform using Reagent kit version 2. Both library preparation and next-generation sequencing were performed by the NC State University Genomic Sciences Laboratory (Raleigh, NC, USA). Raw data can be retrieved from the Short Read Archive (SRA) under the Bioproject accession number: PRJNA905450.

No sequencing was performed for any endosymbiotic or endophytic bacteria wild-type plasmids due to the stability and integrity of the plasmid DNA during the fragmentation procedure.

#### Host discriminant genomic analysis

The aim of this analysis was to identify potential genes or insertion sequences from bacteria in the cassava genomic DNA samples (genotype Valencia); these sequences were believed to induce gall formation in otherwise healthy plants. To do so, a stepwise filtering strategy was implemented. First, sequence read quality was assessed with FastQC (Andrews, 2010); when needed, index, adapters, and low-quality sequences were eliminated. After quality control, reads from both healthy and gall tissues were mapped against the cassava reference genome CV AM560-2 (Phytozome genome ID: 520; Bredeson et al., 2016) using BBmap, part of the BBTools suite (Bushnell, 2015), with the semiperfect mode option. Unmapped reads from healthy leaf tissue and leaf galls were compared against each other, and the shared reads were filtered using BBduk, part of the BBTools suite. The remaining gall reads, unmapped to the reference genome and unmatched to healthy leaves according to defined parameters, were *de novo* assembled into contigs using SPAdes (Prjibelski et al., 2020). A MegaBlast of the selected contigs was performed, setting the minimum length to 200 bp and the percentage of homology to ≥95%. The resulting contigs, the product of gall exclusive reads, were then mapped against the cassava reference genome using the Burrows–Wheeler Aligner (BWA-MEM) (Li and Durbin, 2009). Alignments with low mapping quality (≥20) were filtered using SAMtools (Li et al., 2009). Contigs with matching sequences in either their 5′, 3′, or both ends but non-matching sequences in their core, based on the CIGAR string, were selected as potential foreign DNA insertion sites. Reads were mapped back to the gall hybrid/fusion contigs containing potential foreign DNA insertion sites to assess coverage. Contigs with less than three cover reads were discarded, except for three of them that showed significant annotations with known gene sequences.

We also analysed the putative gall insertion sequence marker (named gall fragment) experimentally in plasmids isolated from all seven isolated endophytic bacteria and the two bacteria isolated from the larval insect head of *I. brasiliensis* and *in silico* by bioinformatic tools in the assembled genomes of the same sequenced bacteria species. In particular, for the *in silico* analysis, isPCR and the primer sequences designed for the gall fragment marker did not show any amplification from the cassava reference genome, nor did it produce amplification products for the insect endosymbiotic bacteria genomes.

#### Metagenomic sequence analysis and taxonomic profile assignment

Shotgun metagenomic sequencing approach was used to analyse the microbiome associated with cassava galls and healthy leaf tissues. Taxonomic profiles were performed using Kraken2 (Wood et al., 2019) and its standard database, which includes bacteria, archaea, viruses, and eukaryotic genomes (https://benlangmead.github.io/aws-indexes/k2). Analyses were applied to reads data (QC filtered reads) from the sequenced gall and healthy tissue samples with an abundance filter of 10K reads.

Taxonomic profiles were also carried out to identify unique reads in the gall tissue following the host discriminant genomic analysis (HDGA) methodological approach to determine their taxonomic origin. Reads were mapped against the cassava genome, retaining only unmapped reads. We then clustered the sequences to obtain unique reads by sample only. This finding was further confirmed by contrasting the resulting taxonomic profiles of healthy samples with gall samples.

After a detailed analysis of the taxonomic profiles was carried out to identify the likely contaminants introduced during processing or due to any potential contaminant event during the final stage of DNA purification of cassava healthy leaves and gall samples, five bacteria taxa were removed as possible external contamination in all sequenced samples.

#### Bacteria endophytic condition determination by synteny analysis

We applied synteny bioinformatic analysis to confirm whether isolated bacteria from both gall tissue and the inducing insect were components of the endophytic microbiome in this structure. To carry out this analysis, exclusive gall reads and bacteria samples were *de novo* assembled using SPAdes (Bankevich et al., 2012) in their metagenome mode for gall reads and isolate mode for bacteria samples. To confirm synteny, contigs from both sides were compared using BLAST+ (Camacho et al., 2009).

#### Genome annotation and functional analyses

To analyse potential differences in terms of function in bacterial groups (endophytic vs. endosymbiont), an enrichment analysis was performed using the Gene Ontology (GO) terms included in the TopGO R package (Alexa, 2022) and a cluster of gene ontology approach (COG) (Galperin et al., 2019). To carry out the analysis, bacterial genomes previously assembled were annotated using prokka (Seemann, 2014). To obtain their corresponding GO and COG terms, the proteins were further annotated using eggnog (Huerta-Cepas et al., 2019). To obtain the raw counts of genes, original assembly reads were mapped back to the annotated genes using BWA MEM (Li, 2013) and filtered with SAMtools (Li et al., 2009), including flags 0 × 8 for non-paired reads and 0 × 2 for properly paired reads. Then, raw counts were obtained using the HTSeq-count from the HTSeq framework (Anders et al., 2015). Finally, differential abundance analysis was performed on shared genes using DESeq2 (Love et al., 2014), allowing their differentiation in abundance post-analysis of their GO terms.

#### *Ab initio* predictions

*Ab initio* prediction was performed on the differentially amplified fragments from *M. esculenta* gall tissues (consensus DNA sequence) and on amplified fragments from purified plasmids of the endophytes IEB 1-2, IEB 3-1, and IEB 5-1, as well as purified plasmids from ISB 1 and ISB 2 isolated from the larval head of the inducing insect. These fragments were amplified with specific gall primers as previously described in the PCR methodology section. *Ab initio* prediction was also carried out on the hybrid sequence fasta files. Gene predictions and their functions were performed using the pipelines of Rapid Prokaryotic Genome Annotation (Prokka) (Seemann, 2014) on the web server Galaxy (www.usegalaxy.org). The pipeline of Prokka uses Prodigal and UniProt for the prediction of coding regions and functional similarity, respectively.

#### Identification of gene segments

The identification of conserved genetic regions in all target sequences obtained by PCR and in the possible hybrid sequences of interest, obtained by high-throughput sequencing, was performed using the PipMaker program (http://pipmaker.bx.psu.edu/cgi-bin/pipmaker?advanced) to produce local alignments of the genetic sequences using BlastZ (Schwartz et al., 2003), and dot plots of the gap-free segments of the alignments were generated as unbroken diagonal lines, indicating a high degree of identity across the genetic sequences (Schwartz et al., 2000).

### Inoculation assays for primary gall induction

Two bacteria grown in solid and liquid YEB 1× culture medium (Piñol et al., 1996) were used to inoculate emerging leaves from apical buds and young leaves of *M. esculenta* (Cassava). Bacterial strains isolated from the larval head of *I. brasiliensis*, subsequently identified as *Rhodococcus* (related to *Rhodococcus* sp. P-2 and *Rhodococcus erythropolis* species, according to the taxonomic profile), were cultured in YEB 1× solid medium and cultured at 26 ± 1°C in a shaker to 110 rpm (Hotech® model 721-2T, Hotech Instruments Corp., New Taipei City, Taiwan) for 3–4 days in the dark. Subsequently, a single clone was used to inoculate 5 mL of YEB 1× liquid medium. After culturing under shaking conditions at 26 ± 1°C for 2–3 days in the dark, a small bacterial suspension was transferred to 10 mL of YEB 1× liquid medium and transferred once again under a sterile biosafety flow-hood to YEB 1× solid medium and cultured for 2–3 days under the above conditions. Then, a single clone was used to inoculate 100 mL of YEB 1× liquid medium to the same preconditions. The endophytic bacterium *Pantoea ananatis*, one of the endophytic bacteria isolated from gall tissue, was used as a control bacterium. The management and culture of this bacterium were carried out under the same conditions described above.

*In vitro* micropropagated *M. esculenta* plants were used for bacterial inoculation assays under sterile conditions. Apical bud and leaf sections of 0.5–1.0 cm were used for inoculation. Explants were maintained first in solid MS medium for different periods of time. Prior to plant material co-cultivation with bacterial cultures, all explants were treated with slight mechanical abrasion using wet filter paper impregnated with ground glass. Just before inoculation, bacterial biomass was measured with a Lambda 25 spectrophotometer (Perkin Elmer-Applied Biosystems, Foster City, California, USA) and set to a OD_600_ value of 0.5–1.0. Then, *in vitro* plant material was submerged into bacteria cultured in YEB 1× liquid medium and shaken in the dark at 28 ± 1°C, for 16–24 h. Inoculated explants were placed in sterile glass Erlenmeyer flasks and washed three times with sterile water. Excess water was removed, and explants were grown separately on MS solid (agar 5.7 g/L) or liquid medium without plant growth regulators. Half of the cultures were supplemented with 3 mg L^−1^ Kathon (only in solid medium), a bacteriostatic reagent used to inhibit bacterial growth in the cultured medium, under white, fluorescent light with an irradiance of 27 μmol m^−2^ s^−1^ at 28 ± 1°C and 110 rpm in a shaker for the liquid medium (Hotech® model 721-2T, Hotech Instruments Corp., New Taipei City, Taiwan). Both types of explants not co-cultured with the bacteria but treated with mechanical abrasion were employed as controls. Thirty to fifty explants were used in each experiment, which was repeated five times in solid medium and two times in liquid medium. Kathon reagent was used only in the first assay carried out in a solid medium.

Inoculation of plants grown in soil under controlled greenhouse conditions was also attempted. The experimental conditions and bacterial growth were carried out using the same methodology. Groups of 50 plants per treatment grown in pots with soil were used to manually inoculate one to two new emerging leaves. Before bacterial inoculation, the leaf surface was carefully cleaned with 70% ethanol. Then, the emerging leaves were treated with a slight mechanical abrasion using a sterilised YEB 1× liquid medium containing ground glass, followed by rubbing the leaf surface by hand using clean latex gloves. After this, sterilised cotton impregnated with liquid medium containing the bacterial growth was placed and fixed on the prepared leaf surface for 1–2 days. The inoculated leaf area was covered with sterile gauze and sterile insulating plastic. This inoculation test was repeated three times.

A randomised block design was followed in this assay. However, because of the viability decline observed in cassava leaves under the established experimental conditions, only presence–absence results for gall-like structure and abnormal tissue development were displayed graphically. Infostat software version 2020p (http://www.infostat.com.ar) was employed to graph the results obtained from the experiments (Di Rienzo et al., 2020). Daily evaluations were performed in all treatments for 6 weeks. Data are shown as the mean and standard error (±SE) of tissue response due to inoculation with the bacteria. Taxonomic identification of the alleged endosymbiotic bacteria strain from the inducing insect (*Rhodococcus*, isolation ISB 2), used in these gall induction assays, was determined before each test by 16S gene sequencing and then confirmed by a taxonomic profile using genomic bioinformatics tools, as also performed for endophytic bacteria.

### Sample preparation for scanning electron microscopy and transmission electron microscopy

Samples of healthy leaves and galls of different sizes from *M. esculenta* plants, as well as the larvae of the inducing insect, were prepared as described by Sánchez et al. (2006), with the modification of fragmentation in liquid nitrogen and dehydration by passage through a gradient of acetone solutions. Sample preparation was performed at the Center for Research in Microscopic Structures, University of Costa Rica, San José, Costa Rica. Samples were observed using a scanning electron microscope (Hitachi S-3700, Tokyo, Japan) with an acceleration voltage of 15 kV.

For transmission electron microscopy (TEM) analyses, samples were fixed with a modified Karnowsky solution, then post-fixed with 2% osmium tetroxide for 1 h, submitted to contrast, and dehydrated in a gradient of acetone solutions (i.e., 30%, 50%, 70%, 90%, and 100%). After washing with 100% acetone, the samples were submitted to pre-infiltration with 1:1 Spurr resin/100% acetone for 5 h with shaking. The subsequent filtration of the samples was performed with pure resin for 12 h. The samples were then shaped and polymerised at 70°C for 3 days. The blocks were then cut into 70-nm-thick sections on an ultramicrotome (Leica Power Tome PC, Leica Microsystems GmbH, Wetzlar, Germany) with a diamond blade (45°), mounted on uncoated 200-mesh copper grids, and stained with uranyl acetate and lead citrate. TEM observations were performed using a Hitachi model HT-7700 microscope (Tokyo, Japan) operating at 100 kV.

## Results and discussion

### Characterising a potential gall molecular marker

To assess our hypothesis, DNA samples from healthy leaf and gall tissues from the same plant were purified (**Figures 1A, D1–D8**). Samples were collected from healthy leaves of *M. esculenta* and galls induced by Cecidomyiidae *I. brasiliensis* (**Figures 1B, C**), and these were carefully cleaned and sterilised to guarantee total epidermis disinfection, including the inner gall chamber.

Possible exogenous DNA, or a DNA insertion sequence present only in gall cells but not in the healthy plant tissue of *M. esculenta* plants, was initially explored using specific PCR primers as a potential gall marker. The primer pair was designed based on the consensus DNA sequence resulting from the alignment of differentially amplified fragments obtained through a previously carried out modified RAPD assay.

A high number of RAPD assays were performed using different random primers; however, decamer primers derived from conserved sequences of the ipt and iAAM genes harboured in the transfer DNA of the Ti plasmid of *Agrobacterium* species and other related bacteria generated the highest number of differentially amplified fragments from gall samples. The quality of purified DNA, as well as the modified RAPD amplification conditions and thermal conditions, allowed us to obtain high reproducibility and reliability in the DNA profiles at different concentrations of analysed DNA. Analytical detections of the RAPD amplicons by gel electrophoresis and the Microchip Electrophoresis System for DNA/RNA (MultiNA) showed differentially amplified fragments of several sizes in all gall samples tested, often from 100 to 4,500 base pairs (bp) (**Figures S3B, C**). Using these approaches, we isolated and obtained the nucleotide sequences of four samples from the most common differentially amplified fragments derived from gall genomic DNA. Three of these sequences were identical, showing the same nucleotide sequence as the fragment of approximately 500 bp (**Supplementary Data 1**). The consensus DNA sequence obtained was used as a template to design and test specific PCR primers. For further information, see **Supplementary Text**.

**Figure 2:**
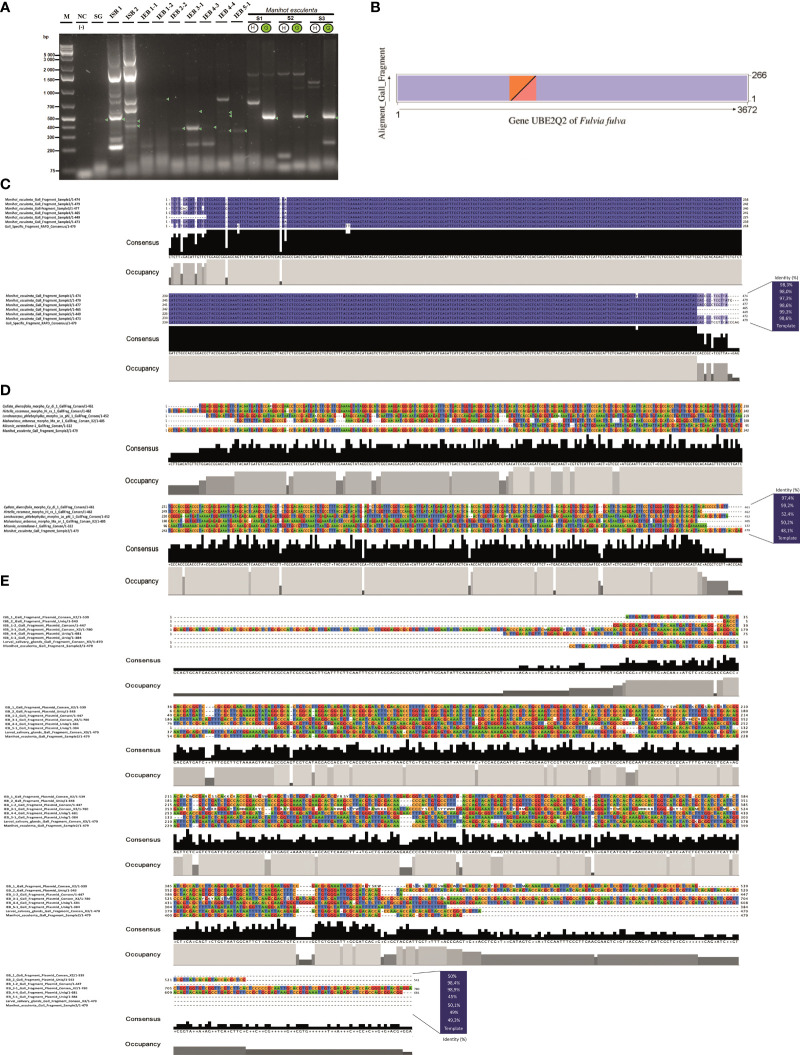
Genetic characterisation of the specific gall fragment marker. **(A)** Gel electrophoresis of PCR products using primers for the specific gall fragment marker (SGF) from purified wild-type plasmids of two putative endosymbiotic bacteria, *Pseudomonas* and *Rhodococcus*, isolated from the larval head of the inducing insect *Iatrophobia brasiliensis* (isolates ISB 1 and ISB 2), as well as from purified wild-type plasmids of seven possible endophytic bacteria isolates selected from the cassava plant gall tissue (IEB). PCR amplicons are also shown for the inducing insect salivary gland sample (SG). Samples of DNA purified from healthy leaf and gall cassava tissues were used as positive reaction controls (lines S1–S3). Lane M, molecular weight marker (Gene Ruler 1 KB Plus); line NC, negative control (reagents only). Green circles indicate positive samples for the specific gall fragment amplification (S1–S3). **(B)** Dot plot representation of the aligned and annotated specific gall fragment sequence showing overlapping regions with the *UBE2Q2* gene of *Fulvia fulva*. The overlapping region between the sequences is shown as coloured triangles in each of the represented axes. **(C)** Alignments of specific gall fragments amplified and sequenced from six gall samples compared to the 479 consensus bp DNA reference sequence. **(D)** Alignments among the 479 consensus DNA sequences of the specific gall fragment from cassava and five sequenced gall morphotypes of different host plant species. **(E)** Alignment of sequenced PCR amplicons performed using specific gall fragment primers over purified wild-type plasmids of two alleged endosymbiotic bacteria isolated from the larval head of the inducing insect *I*. *brasiliensis* (colony-forming units ISB 1 and ISB 2) and from purified wild-type plasmids of seven possible endophytic bacteria isolations selected from the cassava plant gall tissue (colony-forming units IEB), as well as from PCR sequenced fragments from the inducing insect salivary gland (SG). The consensus sequence of the specific gall fragments (SGF) of cassava was used as a template sequence. Grey bar plots show the occupancy within each sequence position, and the black bar shows the base consensus within each sequence position in the resulting alignment.

The gall DNA fragment differentially amplified from gall samples (which we call a specific gall fragment, SGF) was isolated and sequenced. The expected PCR fragment was specifically amplified only in gall samples from *M. esculenta* (**Figure 1E**), total DNA from the inducing-insect salivary glands, and in the isolated wild-type plasmids from two putative insect endosymbiotic bacteria of the genera *Rhodococcus* (ISB 2 bacterial isolate) and *Pseudomonas* (ISB 1 bacterial isolate) (**Figures 2A**, **S4**). Moreover, PCR products with sizes between 350 and 600 bp were also amplified from wild-type plasmids of all endophytic bacteria isolated from cassava gall tissue (**Figures 2A**, **S4**). Additionally, specific gall PCR fragments showing a size similar to that expected from cassava galls were selectively amplified from gall morphotype samples of eight different host plant species (**Figure 1F**). Many of these fragments were sequenced and then aligned to the consensus sequence of the differentially amplified fragment from the cassava gall. The aligned sequences showed high identity with the reference DNA fragment in all *M. esculenta* gall samples (99%–100%) (**Figure 2C**) and with the two IEB isolates 1-2 isolated from inner plant gall tissue (98.9%) and ISB 2 isolated from the larval insect head (98.4%) (**Figure 2E**). Furthermore, samples of other galls from *Cydista diversifolia* (97.4%) and *Hirtella racemosa* (99%) also showed high identity (**Figure 2D**), but a less conspicuous homology (between 48% and 53%) was detected from galls on *Lonchocarpus phlebophyllus*, *Malvaviscus arboreus*, and *Miconia oerstediana* (**Figure 2D**). This, in turn, suggests a diversification of the coding genetic element under different selection pressures for functional adaptation. Moreover, a repeatedly amplified DNA fragment from the salivary glands of a similar size to that expected showed an identity mean value comparable to the previous reference (49.3%), which could suggest that other bacteria in the salivary glands or an insect homologous gene might harbour a similar DNA sequence (**Figure 2E**).

A real-time PCR marker by the TaqMan probe was designed and tested following a similar approach used for end-point PCR assays with corresponding modifications. The results showed amplification signals only in the gall tissue (**Figures 1G, H**) and in plasmids purified from the isolated colonies of the insect endosymbiotic bacteria *Pseudomonas* sp. and *Rhodococcus* sp. (results not shown).

The differentially amplified gall DNA fragment from gall samples (SGF) did not show statistically significant similarity with any other reported gene according to the Basic Local Alignment Search Tool [BLAST, NCBI Genbank database, and Integrated Microbial Genomes & Microbiomes (IMG/M) system, https://img.jgi.doe.gov] and annotation analysis. Nevertheless, several results showed partial pairings with low to medium and often discontinuous length coverage with some ubiquitin-like genes, more specifically, the ubiquitin-like gene *E2*, a component of the ubiquitin-proteosome system (UPS), which has frequently been reported in different fungus species. This fragment showed partial homology to the Ubiquitin-conjugating enzyme E2 Q2 of the fungus *Fulvia fulva*, which was among the most significant (E-value = 6.37e−29, identity = 71.8%, 266 bp of length, accession number CP090172) (**Figure 2B**). However, we predicted that it was different enough to represent a new ubiquitin-like regulatory genetic element associated with the manipulation of the ubiquitin–proteasome system, used indirectly by the inducing insect via the bacteria to manipulate and redirect plant development during gall formation.

The ubiquitin gene family encodes peptides involved in protein–protein signalling and destination as a component of the basic cellular regulation machinery. These types of proteins regulate gene expression at the transcriptional (Adams and Spoel, 2018) and post-translational levels (Xu and Xue, 2019; Liu et al., 2020). The ubiquitin gene family has been reported as an essential part of molecular cell manipulation mechanisms in different pathogen and endosymbiotic–host interactions (Janjusevic et al., 2006; Vierstra, 2009; Park et al., 2012; Singer et al., 2013; Banfield, 2015; Kud et al., 2019). Ubiquitin-like genes from bacterial secreted effector molecules with structural and/or functional similarity to UPS pathway components mimic and modify the host UPS system (Ramachandran et al., 2021), allowing the hijacking of the cellular machinery, as has been previously reported in the crown gall system induced by *A. tumefaciens* (Magori and Citovsky, 2012; Lacroix and Citovsky, 2015). The UPS system is considered the major protein turnover pathway found across all domains of life and is especially important in regulating almost all plant development signalling pathways, including hormone-mediated plant growth and development, as well as plant responses to stress (Santner and Estelle, 2010; Sadanandom et al., 2013; Shu and Yang, 2017; Adams and Spoel, 2018; Xu and Xue, 2019). Although less is known about UPS manipulation in galling insects, there is emerging evidence that plant development can be manipulated through the UPS system by effector molecules in the salivary glands of the gall inducer (Zhao et al., 2015).

### Genetic insertion events inferred from bioinformatic analysis provide evidence for plant cell transformation

**Figure 3:**
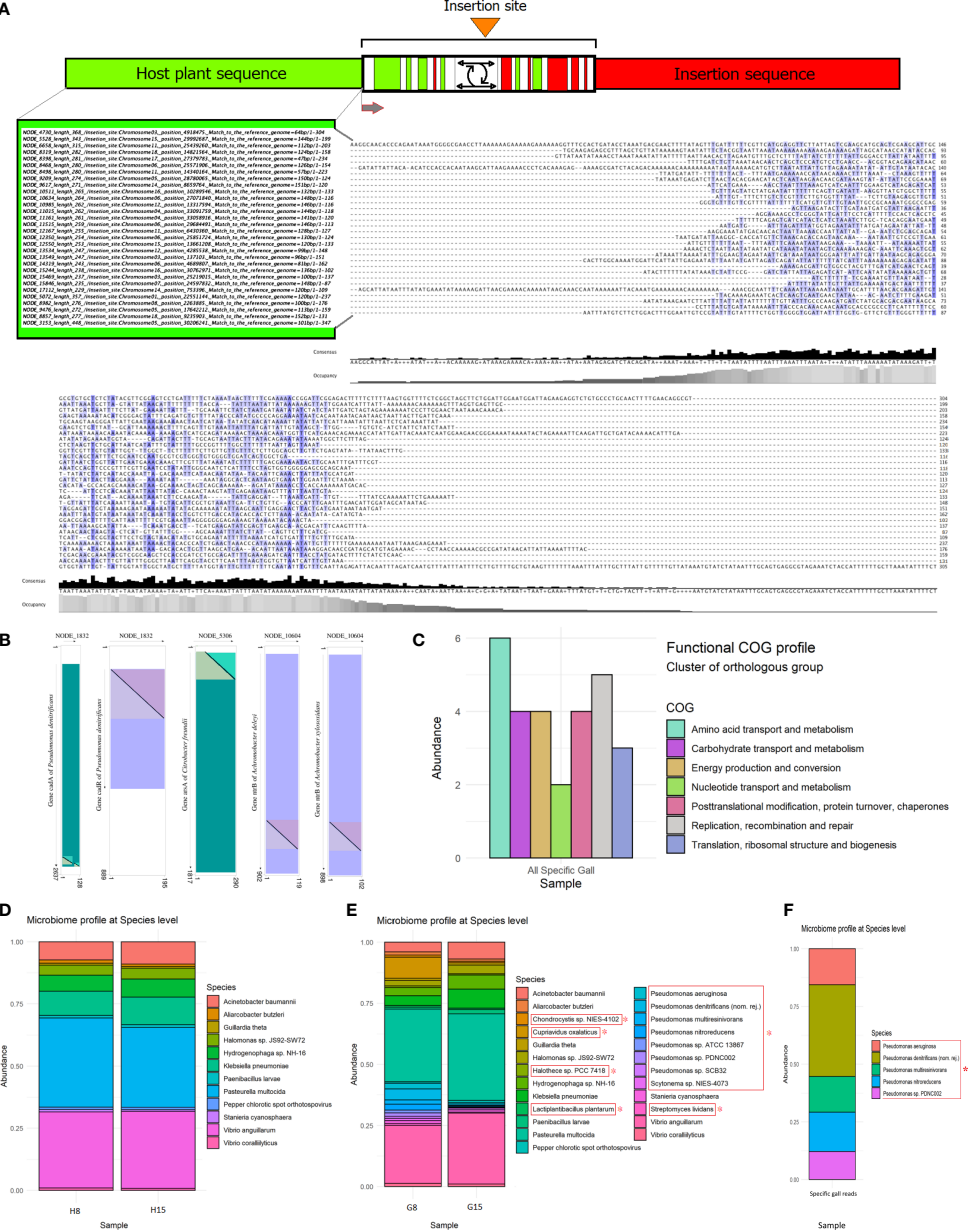
Bioinformatic analysis expands the catalogue of gall-specific sequences. **(A)** Theoretical diagram of the insertion regions according to the methodological approach applied. Position of the insertion sites into each host chromosome in the *Manihot esculenta* reference genome is shown on the left in a selected subgroup of hybrid contigs, mostly in forward orientation. Only the alignment framework of unmatched sequences to the cassava reference genome from hybrid/fusion contigs is shown on the right. Grey bar plots show the occupancy within each sequence position, and the black bar shows the base consensus within each sequence position in the resulting alignment scheme. **(B)** Dot plot representation of the aligned and annotated hybrid contigs region of unmatched sequences to the cassava reference genome, showing candidate insertion sequences in several of the hybrid contigs assembled, harbouring known DNA sequences revealing partial significant identity matches and covering with reported genes. Overlapping regions between the contigs and the annotated genes are shown as coloured triangles in each of the represented axes. Purple colour in the referenced gene represents forward strain orientation, and the blue-green colour (viridian) represents reverse strain orientation. **(C)** COG function classification histogram. Count of genes belonging to the COG categories related to exclusive gall reads involved in essential metabolic pathways and biological functions. **(D, E)** Microbiome profile of healthy plant tissue and gall tissue samples. Relative abundance of microorganism taxa identified in the microbiome of healthy leaves and gall samples of cassava. Taxonomic profiles were carried out to a 10K filter using raw reads generated by the shotgun sequencing approach. Each bar represents the organism taxon detected in one sample. The profile showed a similar abundance between both healthy samples **(D)**, but a different relative abundance of microorganisms between gall samples. **(E)** Asterisks indicate enriched or exclusive microorganism species present only in gall tissue according to the taxonomic profile carried out, comparing sequenced heathy leaf samples with gall samples. The most common core endophyte taxa between leaf and gall tissues are also shown. **(F)** Taxonomic identity profile (10K) associated with some of the selected gall-specific reads. Gall-specific reads were bioinformatically filtered from the sequenced gall samples, which mismatched with the cassava reference genome and filtered against shared reads from healthy tissue samples.

Through a discrimination approach using shotgun metagenomic sequencing, we showed the presence of potential foreign-exclusive DNA within plant gall tissue. We named this methodological approach host discriminant genomic analysis (HDGA) (**Figure S5**). HTS data from healthy leaves and gall tissue (16× sequencing depth) were processed to separate, assemble, and analyse the DNA sequence reads different from the referenced host plant genome of *M. esculenta*. On average, 4.6% of the raw reads from both healthy leaf and gall tissues did not map to the reference genome. Furthermore, of those unmapped reads, 12.4% were unique to the gall tissue samples. *De novo* assembly of these specific reads produced 17,148 contigs. Eight of these assembled contigs produced fragments with sizes between 2,000 and 2,501 bases, 306 between 1,000 and 2,000 bases, 2,161 contigs of 500–1,000 bases, 14,664 fragments between 200 and 500 bases, and 8 fragments smaller than 200 bases (**Supplementary Data 2**). Using this approach, reads that did not map to the cassava reference nor were they shared between healthy and gall tissue, represent potential foreign DNA from endophytic organisms unique to galls, such as bacteria or fungi, or possible foreign DNA integrated into the genome of gall cells (see **Figure S5** for the general pipeline-flow diagram approach). The resulting assemblies were compared against the reference *M. esculenta* genome to identify possible hybrid/fusion fragments, which must harbour homologous sequences with the host plant, along with external sequences without any homology to the host reference genome. When the assembled contigs were filtered once again following this approach, 407 contigs were retained, 59 of which had 300–571 bases, and 348 had 229–300 bases (**Supplementary Data 3**). Our analysis found 130 of the reported hybrid/fusion contigs with coverage from 10 to 30 reads. A total of 124 showed coverage ranging from six to nine. Only 74 displayed five to four reads, and 63 had three reads. Moreover, seven contigs showed the largest number of reads, with more than 95 (**Supplementary Data 3**). These hybrid sequences represent potential insertion regions for foreign DNA integrated into the genome of gall cells in the sample of mixed galls used for library preparation. Thirty hybrid contigs showing the highest alignment parameters when compared with the cassava reference genome, mainly mapped to the forward strand of this reference genome, are shown in **Figure 3A**. The estimated insertion position within the assigned chromosomes in the host *M. esculenta* genome is also shown, evidencing multiple possible insertion sites in the genome of plant cells (see **Supplementary Data 3** for all candidate hybrid assemblies).

Structural variations, such as repeated sequences, including retrotransposons (Bartlett et al., 2014), inversions, in tandem sequences (Gang et al., 2019), deletions, duplications, and other complex rearrangements, including vector backbone or chromosome sequences carried over together with insertion sequences, may occur around or near the insertion point regions bordering the exogenous DNA and in the host plant DNA. A similar phenomenon has been reported in the flanking regions of the T-DNA insertion fragment harboured in the Ti plasmid of *A. tumefaciens* (Krizkova and Hrouda, 1998; Brunaud et al., 2002; Bartlett et al., 2014; Kleinboelting et al., 2015). They could also occur due to the activity of transposable elements (Wicker et al., 2016), such as *Mariner-*like elements in the genomes of seven species of *Rhus* gall aphids (Ahmad et al., 2021). The integration sites within plant genomes seem to be largely randomly distributed under non-selective conditions (Kim and Gelvin, 2007). Small areas of microhomology in insertion sites between T-DNA and neighbouring plant genomic DNA have also been reported (Brunaud et al., 2002; Kleinboelting et al., 2015). The analyses of microhomology indicate that this type of sequence could be, most of the time, a prerequisite for integration events (Gorbunova and Levy, 1999). Furthermore, a depth analysis of the host plant flanking sequences revealed a high proportion of the characterised T-DNAs inserted into or close to repetitive elements in transgenic barley lines without causing negative effects on transgene expression (Bartlett et al., 2014).

Because each insertion process is a single event that potentially generates a structurally hypervariable region in the DNA around the specific insertion site, finding a consensus sequence or motif around these sites is a challenge (**Figure 3A**). Those regions containing hypervariable sequences around the integration sites could certainly limit the assembly of reads towards the exogenous DNA sequence, thus restricting the length in hybrid/fusion contigs reported in this survey. Likewise, the extension of read assemblies towards the plant genome orientation in the hybrid contigs is also restricted because all common reads between the sequenced genomes of healthy plants and galls were filtered out, primarily with the reference cassava genome and then by pairing against themselves. The alignment quality with regard to the reference cassava genome, filtering stringency, and the read coverage obtained in our analysis provide evidence in support of the hypothesis that genetic material is inserted into the genomes of plant gall cells (**Figure 3A**, **Supplementary Data 3**). Technical artifacts, such as sequencing errors, formation of chimeric DNA during library preparation, and nonspecific assemblies, are unlikely to account for all our observations of putative hybrid/fusion fragments, nor is the presence of hypothetical orthologues of genes from an endophytic microorganism in the plant genome.

BLAST and annotation analysis of the unmatched sequences in some hybrid contigs not associated with the *M. esculenta* reference genome did not show a high identity frequency with reported DNA sequences, while others showed low identity and coverage. However, following our methodological approach, we located known candidate insertion sequences in several assembled hybrid contigs. Some of these candidates harbour DNA sequences revealing partially significant identity matches and cover with reported genes (**Figure 3B**, **Supplementary Data 4**). Among the outstanding associated genes, transcription regulatory factor CadR and CadA (Cd^2+^ transporting ATPase enzyme) were found in the hybrid contig NODE_1832_length_571_cov_2.279352_0 (E-value = 8E−89, identity = 96%, 194 bp of length, accession number CP043626 and E-value = 6E−33, identity = 81%, 127 bp of length, accession CP043626, respectively) (**Supplementary Data 4**, **Figure 3B**), located at the distal end to the possible insertion site in the region not associated with the cassava reference genome, which showed identity with *Pseudomonas* species, such as *Pseudomonas nitroreducens* strain HBP1, *Pseudomonas denitrificans* strain BG1, and *Pseudomonas multiresinovorans* strain populi, which will be reported later as exclusive or enriched components of the gall microbiome (**Figures 3D, E**). Likewise, significant putative insertion sequences associated with the *arsA* gene (arsenical pump ATPase), NODE_5306_length_349_cov_1.058824_1 (E-value = 9E−112, identity = 100%, 215 bp of length, accession number CP070545), GTP-binding protein encoded by the *obg* gene, NODE_9526_length_272_cov_0.441026_16 (E-value = 1E−12, identity = 76%, 107 bp of length, accession AP018162), nitrate transport permease protein encoded by the *nrtB* gene, NODE_10604_length_264_cov_0.786096_0 (E-value = 4E−46, identity = 92%, 118 bp of length, accession CP065997), assimilatory nitrite reductase enzyme encoded by the *nasE* gene involved in biological nitrate assimilation, NODE_10604_length_264_cov_0.786096_0 (E-value = 2E−44, identity = 91%, 118 bp of length, accession LT976871), and bicarbonate transport system permease protein encoded by the *cmpB* gene, NODE_10604_length_264_cov_0.786096_0 (E-value = 3E−35, identity = 85%, 118 bp of length, accession LR594671) were also found (**Figure 3B**, **Supplementary Data 4**). Interestingly, despite its similarity to reported genes in bacteria, all of them are essential for rapid plant growth, maintenance, and survival under unfavourable conditions and are also associated with gene regulation mechanisms (**Supplementary Data 4**).

Additionally, the association among the 17,148 gall-specific contigs as potential components of an integrated DNA fragment in the gall cells could be inferred based on their annotated function. However, for many hypothetical proteins, a function could not be assigned (**Supplementary Data 5**). The presence of different transcriptional, post-translational, and cell cycle regulatory factors, as well as exogenous polymerases, transposases (transposase A), or the integration host factor subunit alpha of bacteriophage lambda (which plays a crucial role in the insertion process of lambda DNA into the *Escherichia coli* chromosome), among others, within the gall-specific contigs, provide indirect evidence and are particularly revealing.

Functional Cluster of Orthologous Group (COG) profile analysis, using gall exclusive contigs, showed that the COG categories of amino acid transport and metabolism, carbohydrate transport and metabolism, energy production and conversion, nucleotide transport and metabolism, posttranslational modification, the translation of the ribosomal structure and biogenesis, and DNA replication, recombination, and repair were the main functional categories represented. Although a relatively small number of contigs were used due to bioinformatic processing, these results could indicate that functions related to growth, transport of plant metabolites to inner gall tissues, replication, and expression of nucleic acids, in addition to gene regulation at different levels, might be promoted or increased by the exogenous DNA detected in gall cells (**Figure 3C**).

### Metagenomes reveal an enriched microbial community in galls

We used HTS to analyse the metagenomes of two gall samples, each consisting of a pool of galls and two healthy tissue samples. The average percentage of assigned reads to any species-level taxon for healthy tissue samples was 4.64%, whereas 4.77% was the average for gall samples, according to reference databases. We showed only the significant microorganisms found at a filter resolution of 10K. The resulting taxonomic profile showed a common microbiome between gall and healthy tissue samples, including a core community dominated by 12 taxa, 9 of which were bacteria, namely, *Acinetobacter baumannii*, *Aliarcobacter butzleri*, *Halomonas* sp. JS92−SW72, *Hydrogenophaga* sp. NH−16, *Klebsiella pneumoniae*, *Paenibacillus larvae*, *Pasteurella multocida*, *Vibrio anguillarum*, and *Vibrio coralliilyticus*, in addition to two cyanobacteria, *Guillardia theta* and *Stanieria cyanosphaera*, and the Pepper chlorotic spot orthotospovirus (**Figures 3D, E**). Furthermore, an exclusive or enriched microbial community was detected in samples of gall tissues. The taxonomic composition included a community dominated by eight species belonging to the genus *Pseudomonas*, two species of cyanobacteria (*Chondrocystis* sp. and *Halothece* sp. PCC 7418), and three other bacteria species, *Cupriavidus oxalaticus*, *Lactiplantibacillus plantarum*, and *Streptomyces lividans* (filamentous bacterium) (**Figure 3E**). A brief overview of the relevant functional characteristics reported for these microorganisms and their possible role in gall development and maintenance can be found in the **Supplementary Text section**.

From the sequencing data exclusive to gall tissue, up to 69.2% of the reads were unassigned to any taxon. These may correspond to microorganisms not included in the available databases at the time of our analysis, or even indicate the possibility of new species as part of this unique microbial community. Nevertheless, a significant amount of exclusive gall reads was associated with several of the same *Pseudomonas* species reported as exclusive or enriched by metagenomic analysis in gall tissue (**Figures 3E, F**).

### Isolation and genomic analysis of putative gall-inducing symbionts and gall endophytes

**Figure 4:**
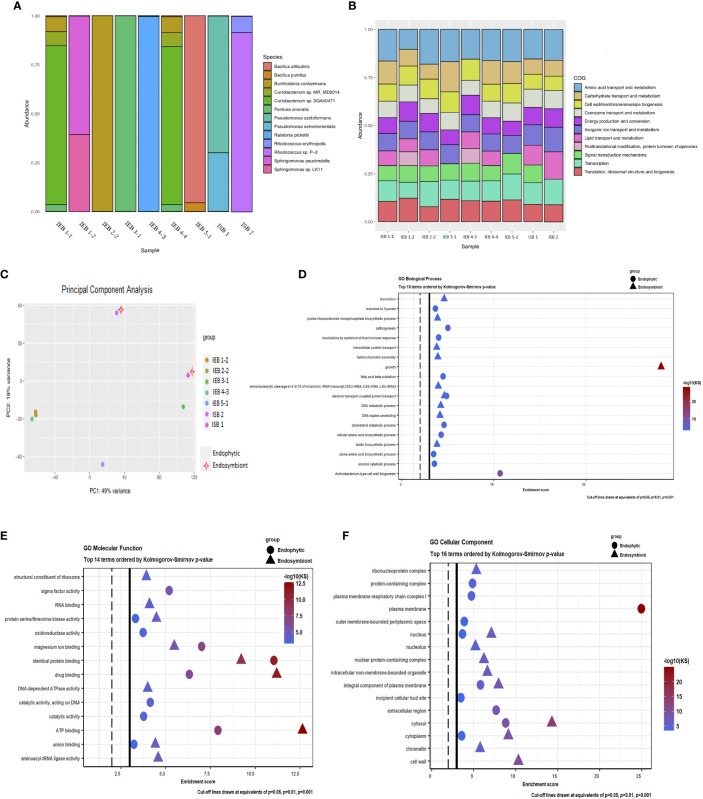
Microbiome taxonomic profile and functional analysis of gall endophytes and putative endosymbionts of the inducing insect. **(A)** Relative abundance of bacterial taxa identified in each of the samples of colony-forming units (CFUs) obtained from gall tissue of *Manihot esculenta* and from the larva head of the inducing insect *Iatrophobia brasiliensis*. The figure displays the most abundant taxa individually, with the remainder grouped together. Each bar represents the bacterial taxa detected in one sample. Each CFU from the insect head was called an isolated symbiotic bacteria (ISB). Each of the CFUs isolated and grown from internal sections of sterilised gall epidermis tissue was called an isolated endophytic bacteria (IEB). **(B)** Distribution of COG functional categories for CFU isolates from gall tissue and from the larval head of the inducing insect. **(C)** Genetic identity comparison of sequenced bacterial isolates classified up to genus by principal component analysis. **(D–F)** Functional analysis showing the top Gene Ontology enriched pathways of endophytic and alleged endosymbiont bacteria genomes. A high score indicates a high degree of enrichment.

Two isolates (CFUs) from the original culture grown from the larval head of the inducing insect, *I. brasiliensis*, as well as seven endophytic bacteria isolates selected from cassava gall tissue, were sequenced using high-throughput platforms. Taxonomic profile analysis applied to all isolates revealed that four corresponded to a single species (IEB 2-2 = *Burkholderia contaminans*, IEB 3-1 = *P. ananatis*, IEB 4-3 = *Ralstonia pickettii*, and IEB 5-1 = *Bacillus altitudinis*). Moreover, three isolates showed association with two bacteria species of the same genus (IEB 1-2 = *Sphingomonas* sp. LK11 and *Sphingomonas paucimobilis*), and the two isolates from the original culture grown from the inducer insect, isolate ISB 1, which was *Pseudomonas azotoformans* and *Pseudomonas extremorientalis*, and isolate ISB 2, which was *Rhodococcus* sp. P-2 (which could not be classified to the species level, major component) and *R. erythropolis*. Likewise, isolates IEB 1-1 and IEB 4-4 were assigned to the same bacteria species: *Burkholderia contaminans*, *Curtobacterium* sp. MR_MD2014, *Curtobacterium* sp. SGAir0471, and *P. ananatis* (**Figure 4A**). However, none of the bacteria isolated from either the gall or the inducing insect larva were precisely detected in the resulting taxonomic profiles belonging to the sequenced samples of healthy plants and gall tissues (**Figures 3D, E**). Nevertheless, through synteny comparison analysis using specific gall contigs as target sequences compared to each of the sequenced bacterial genomes, the endophytic condition was determined only for the bacteria species isolated from gall tissue, not for those from the inducing insect (**Figure S6**). Thus, these endophytes might be a marginal component of the gall microbiome, but many of them could also be exclusive components of the microbial community within gall tissues. None of the gall-specific fragment consensus sequences (obtained experimentally) shared homology or aligned with any of the bacterial genomes. The isolation, identification, and subsequent characterisation of microbiome components are difficult tasks due to their low abundance in tissue. This is especially true when using whole-host genome sequencing techniques. However, this barrier may often be overcome using different culture media for growth.

Interestingly, Yang et al. (2021) reported the same bacterial genera sequenced in this study (both gall and inducing insect), except for *R. pickettii*, in their bacterial community of *Lithosaphonecrus arcoverticus* (Hymenoptera: Cynipidae) and in their gall (galled twigs) in *Lithocarpus glaber* (Fagaceae). *Pseudomonas* and *Sphingomonas* have also been identified as members of a common core bacterial community in willow-galling sawflies (Michell and Nyman, 2021). Moreover, *R. erythropolis*, *P. ananatis*, and *Pseudomonas* spp. were recently identified from the bacterial communities by Yang et al. (2022) as predominant species in chestnut tree galls induced by *Dryocosmus kuriphilus*. These bacteria were also identified as components of the microbial communities of the inducing insect and in *Torymus sinensis*, a host-specific parasitoid of *D*. *kuriphilus*.

Microbial genome comparison by COG functional categories is shown in the COG profile for each isolated CFU (**Figure 4B**). Also, genetic identity comparison by principal component analysis of sequenced bacterial isolates is shown in **Figure 4C**. Gene Ontology enrichment analysis of endophytic bacteria genomes showed that the most relevant enriched terms were those related to *Actinobacterium*-type cell wall biogenesis in the category of biological processes and plasma membrane in the category of cellular components. Of the 14 most significant molecular functions identified, sigma factor activity, magnesium ion binding, identical protein binding, drug binding, and ATP binding were the most significantly enriched. Moreover, growth factors for biological processes, in addition to the nucleus, intracellular non-membrane-bounded organelle, nuclear protein-containing complex, integral components of the plasma membrane, cytosol, cytoplasm, and cell wall of GO cellular component, were significantly increased in putative larval endosymbiotic bacteria. Similarly, magnesium ion binding, identical protein binding, drug binding, and ATP binding were the most significant molecular functions in the enrichment values for these bacteria (**Figures 4D–F**). Significant discrete GO terms from endophytic bacteria and possible insect endosymbiotic bacteria are shown in **Supplementary Data 6**. Thus, functional analyses carried out on the endophytic bacteria and on the presumed endosymbiotic bacteria isolated and sequenced from the gall tissue and insect head, respectively, as well as functions associated with the exclusive or enriched microbial community identified in cassava galls by metagenomic analysis (**Figure 3E**), suggest that the microbial community may play an important role in gall induction, growth, and maintenance. The inferred metabolic pathways and biological functions show that several endophytic bacterial species have a range of different potential functions, including biodegradation of phenolic and potentially harmful metabolic compounds, nutrient supplementation, synthesis of plant hormones, and secondary metabolite degradation. Moreover, this endophytic bacterial community has the potential to synthesise essential amino acids and vitamins, and some of them could be involved in nitrogen and phosphate metabolism, suggesting that these bacterial species could contribute significantly to the nutritional quality of gall tissue. Hence, from an ecological, evolutionary, and functional point of view, our data show that some components of this microbiome can play important roles, both in the host plant itself and in galls. Furthermore, we argue that an unrevealed, induced microbial community that seems to be evident in our findings might have a critical impact on insect gall induction and maintenance despite their low abundance in the gall tissue.

### Artificial gall induction by potential bacterial symbionts

**Figure 5:**
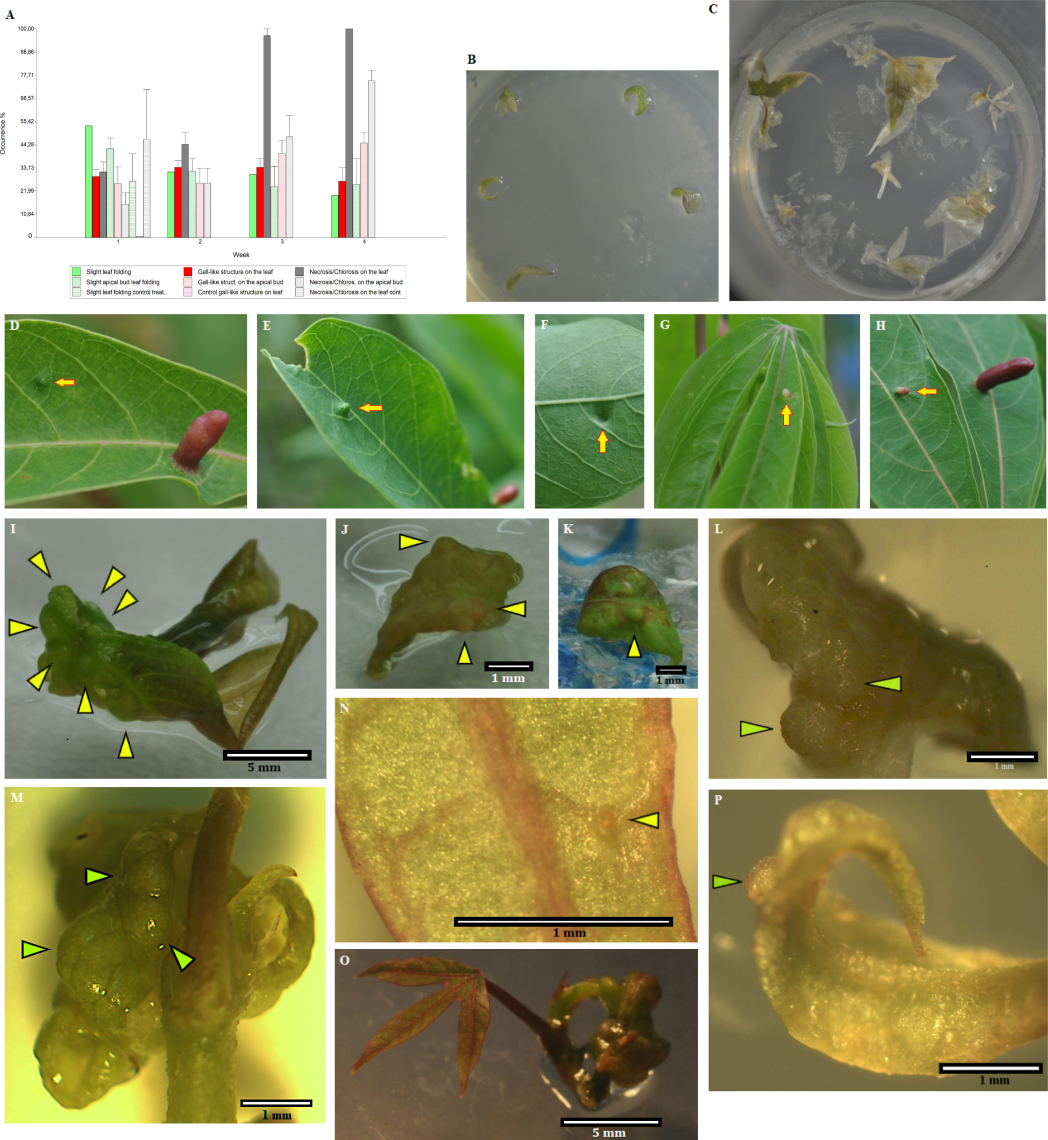
Bioassays show that potential insect endosymbiotic bacteria of the genus *Rhodococcus* induce gall-like structures in *Manihot esculenta* plants. **(A)** Graph of primary gall induction on leaves and micro-stakes with apical buds. Data for bacterial inoculation control are not shown due to the tissue damage caused since the first week of data collection. **(B)** Control inoculation, with only slight mechanical abrasion (without bacteria inoculation) in solid medium. **(C)** Leaf and apical buds control culture, inoculated with *Pantoea ananatis* (IEB 3-1) control bacterium. **(D–F, H)** Initial gall formation induced under natural field conditions on medium-mature young leaves. **(G)** Gall induced under natural field conditions on young leaf primordia. **(I–L)** Gall-like structure induced on leaves by inoculation with the isolated *Rhodococcus* strain (1–2 weeks of culture). The solid medium was supplemented with 3 mg L^−1^ Kathon, a bacteriostatic reagent used to inhibit bacterial growth in the culture medium. **(M–P)** Gall-like structure induced on apical buds by inoculation with the isolated *Rhodococcus* strain (**M**, 4 weeks of culture; **N–P**, 3 weeks of culture). Green or yellowish arrows show the formation of gall-like structures. Data are shown as the mean (± SE) of tissue response due to inoculation with the bacteria.

An initial gall-like structure was obtained using the *Rhodococcus* bacterial strain, which was related to the gall induction process (**Figures 5A, I–P**). Putative insect endosymbiotic bacterial lines isolated from the inducing insect (larva head), initially classified as *Rhodococcus* sp. by sequencing the 16S gene and then identified as the genus *Rhodococcus* based on the taxonomic profile (related to *Rhodococcus* sp. P-2 and *R. erythropolis*), were used in these assays. The endophytic bacterium *P. ananatis* isolated from gall tissue was used as a control bacterium.

Plant tissue culture inoculation assays on *M. esculenta* were carried out under controlled laboratory conditions. Most of the gall-like structures were formed in leaves and leaf sections of *M. esculenta* rather than in micro-stakes with apical shoots (apical buds with small leaf primordia), where their formation was the lowest in the first 2 weeks. However, a greater increase in tissue necrosis was observed after 2 weeks of culture (**Figure 5A**). Moreover, since the culture medium used was not optimised for the propagation and maintenance of leaves, it was not possible to maintain the structure for more than 4–5 weeks. Necrosis and early fall of the inoculated leaves did not allow long-term monitoring of *in vitro* materials (with a few exceptions), an effect also observed in inoculated greenhouse plants.

The inoculation of plants grown in soil under greenhouse conditions was also attempted. However, except for the rare event of neoplasm formation over the inoculated leaf primordia, no gall-like structures were clearly observed (results not shown). This could be the consequence of an increased level of control by the plant in its growth process or unaccounted for environmental factors. Moreover, strong tissue chlorosis was observed after the first week, when the bacteria *P. ananatis* was used in the assay (control inoculation bacterium), observing broad bacterial growth on the tissues without tissue folding or gall-like structures (**Figure 5C**). Likewise, no significant tissue folding or gall-like structure was observed in the control material treated only with mechanical abrasion using the ground glass (**Figure 5B**). The different success rates of inducing gall-like structures in the *in vitro* assays and under controlled greenhouse conditions indicate that specific inoculation conditions have yet to be optimised.

After inoculation and incubation with cassava explants, bacterial colonies on plant tissue were frequently observed and were usually associated with invaginations and folds of the leaf blade. However, these leaf deformations and invaginations were not frequently associated with macroscopically visible bacterial growth (**Figures 5I, K, L, N, P**). Furthermore, a few showed differentiated neoplastic tissue that visually differed from the surrounding tissue (**Figures 5l, N, P**). An increased tissue reaction was observed when a high bacterial density grew over the plant tissues. This increased reaction induced a higher rate of deformation and invagination of the leaf blade, which usually triggered an earlier necrosis condition in the explants (**Figure 5A**, images not shown), probably generated by the hypersensitive reaction of the plant tissue. Initial gall formation induced under natural field conditions on young leaf primordia and on medium-mature young leaves is showed in **Figures 5D–H**.

The ability to induce abnormal gall-like growth has also been reported in *Rhodoccocus fascians* (Stes et al., 2011; Dolzblasz et al., 2018; Harris and Pitzschke, 2020). The development of plant growth is closely related to a linear virulence plasmid harbouring an array of cytokinin genes encoded by the fasciation (fas) operon in most pathogenic isolates (Francis et al., 2012; Creason et al., 2014; Radhika et al., 2015; Jameson et al., 2019). Furthermore, despite the absence of authentic leafy galls in Pistachio Bushy Top Syndrome (PBTS), synergistic coinfection has been reported between *Rhodococcus corynebacterioides* and *R*. *fascians* by Vereecke et al. (2020).

Bacterial strains belonging to the genus *Rhodococcus* (**Figure 4A**), isolated from the inducing insect (ISB 2 isolate) and used in gall induction assays, were not detected with certainty at the species level in the sequenced samples of galls and healthy plant tissues when metagenomic analysis was applied (**Figures 3D–F**). Therefore, we suggest three possible explanations for our findings. The first scenario proposes that the bacterial lines used in our gall induction assays were not conclusively detected because the bacteria had a lower relative abundance within the gall tissue. Thus, the target bacterial DNA would not have a representative fraction in the gall samples purified for sequencing. An additional explanation could be that the putative bacterium is involved in the gall induction process, but it is not a component of the gall cell endophytic microbiome, according to our results, thus performing its action externally to the plant cell. Furthermore, the bacterium could exert its action at the initial stage of gall induction. Third, another taxonomically related bacterium sharing a similar molecular mechanism might also induce the formation of this structure in its initial stage.

### Detection of the specific gall marker fragment in sequenced plant material and sequenced bacterial isolates

The non-detection of the differentially amplified gall fragment (specific gall fragment experimentally obtained) in the sequenced *M. esculenta* gall genome and specifically in the sequenced *Rhodococcus* spp., which we argue is related to the gall induction process, could be explained by the sequencing of large low-copy-number wild-type plasmids harbouring large duplications, making it nearly impossible to correctly determine a plasmid genome sequence using a short-read sequencing platform, such as Illumina HiSeq and MiSeq (Smalla et al., 2015; Orlek et al., 2017). Moreover, large wild-type plasmids are difficult to reconstruct from whole-genome sequencing data; this arduous task usually requires a hybrid assembly approach that combines the long reads with the accuracy of short-read sequencing (Berbers et al., 2020). Consequently, localising genes in specific plasmids may be difficult (Orlek et al., 2017). Hence, based on our findings, no direct evidence that the specific gall fragment could be part of an insertion sequence was obtained, and we therefore argue otherwise that this specific gall fragment would be an accessory genetic component of the bacteria transformation machinery, harbouring a transformation plasmid, but this would not be part of the insertion sequence integrated into the genome of the plant cell, analogous to the configuration of the Ti plasmid in *A. tumefaciens* strains (Suzuki et al., 2015; Shao et al., 2018; Chou et al., 2022). Therefore, its specific detection in the DNA extracted from gall tissue by PCR-based methodologies could be the consequence of the transferred endophytic form of *Rhodococcus* spp. by the inducing insect, or another bacterium sharing the same genetic element, in which genomic and plasmid DNA are co-precipitated along with host plant DNA. Moreover, *in silico* PCR analysis of the specific gall fragment did not produce any amplicon from the cassava reference genome (results not shown), thus demonstrating that this DNA fragment is not a component of the host plant genome. However, further research on this specific topic should be carried out to provide more evidence.

## Conclusion

We provide evidence suggesting an insect-induced gall formation mechanism mediated by genetic transformation events in host plant cells. Our data allude to a potential mobile genetic element harboured in *Rhodococcus* spp. bacteria isolated from the inducing insect that could be involved in the induction mechanism. Moreover, gall induction and growth could also be associated with a change in the microbiome composition in plant tissue. It is possible that the inducing insect injects components of the gall-specific endophytic community during female oviposition and larval feeding. Genetic transformation and microbiome modification of gall tissue in *M. esculenta* could be a more widely distributed induction mechanism in nature. This was supported by the detection of a potential accessory genetic component of the transformation machinery (ubiquitin-like gene *E2*) or with a similar identity in other insect galls in other plant species, and even detected in wild-type plasmids purified from endophytic bacteria species isolated from the same cassava gall tissue. While our results provide greater insight into plant–bacteria–insect interactions, further research is needed to fully understand the complex mechanisms of gall induction and formation. Moreover, the insertion of genetic elements from a putative bacterium could function as a switch in the molecular interaction between the inducing insect and the host plant.

## Data availability statement

The datasets presented in this study can be found in online repositories. The names of the repository/repositories and accession number(s) can be found in the article/**Supplementary Material**.

## Ethics statement

The manuscript presents research on animals that do not require ethical approval for their study.

## Author contributions

OG-B: conceived the survey; substantial contribution to the concept and design of the bioinformatic approach; performed most of the molecular biology experiments; contribution to data analysis and interpretation; contribution to the data collection, registration, and processing; obtained and provided financial support; and wrote the article. JJ-M: substantial contribution to the concept, design, and analysis of the bioinformatic approach; participated in writing the manuscript. RW: contribution to the design and analysis of the bioinformatic approach; and critical revision of the manuscript. PH: contribution to the concept and design of the study; contribution to critical revision; and addition of intellectual content. SV-D: contribution to bioinformatic analysis and interpretation; contribution to critical revision and adding intellectual content; and contribution to manuscript preparation. AA-G: contribution to data analysis and interpretation; and contribution to manuscript preparation. AP-T: contribution to critical revision; adding intellectual content; and contribution to manuscript preparation. All authors contributed to the article and approved the submitted version.
