# Supplementary material for: Microbiome and plant cell transformation trigger insect gall induction in cassava
Source: Front Plant Sci. 2023 Nov 29;14:1237966. doi: 10.3389/fpls.2023.1237966 (PMC10731979; doi:10.3389/fpls.2023.1237966)
Supplement: Supplementary file 1 [file DataSheet_1.zip › Supplementary Material/Supplementary_Text.pdf]

Supplementary text for
Microbiome and Plant Cell Transformation Trigger Insect Gall Induction in Cassava

Supplementary Methods

Galling insect model

The morphotypes of galls include: *Cydista diversifolia* (Kunth) Miers (Bignoniaceae) morphotype Cy_di_1, induced by a Cecidomyiidae; *Malvaviscus arboreus* Dill. ex Cav. (Malvaceae) morphotype Ma_ar_1, induced by a Cecidomyiidae; *Hirtella racemosa* Lam. (Chrysobalanaceae) morphotype Hi_ra_1, induced by a Cecidomyiidae; *Pisonia macranthocarpa* (Donn. Sm.) Donn. Sm. (Nyctaginaceae) morphotype Pi_ma_4, induced by an unkonwn insect; *Randia monantha* Benth. (Rubiaceae) morphotype Ra_mo_1, induced by an unknown insect; *Lonchocarpus phlebophyllus* Standl & Steyerm. (Fabaceae) morphotype Lo_phl_1, induced by a Psyllidae; recorded and described by Gätjens-Boniche et al., 2021. In addition to the gall morphotype on *Coussarea hondensis* (Standl.) C.M. Taylor & W.C. Burger (Rubiaceae), induced by an unknown insect. These galls were recorded and described by Gätjens-Boniche et al. (2021) and the host plants were identified by Alonso Quesada (Herbario del Museo Nacional, San José, Costa Rica). The gall morphotype on *Miconia oerstediana* (O. Berg ex Triana) Michelang. (Melastomataceae), previously identified as *Conostegia oerstediana* and induced by a Cecidomyiidae, was also included; the host plant was identified by Mario Blanco Coto at Herbario de la Escuela de Biología, Universidad de Costa Rica.

Quantification of PCR inhibition level from genomic DNA purified using the specific endogenous gene UBQ-10 of *Manihot esculenta*

Strong PCR inhibition was previously detected in DNA extraction from cassava plant material using different genomic DNA purification protocols and commercial kits. For that reason, PCR reaction conditions were carefully tested. PCR inhibition was tested using the endogenous gene UBQ10 of *Manihot esculenta* according to reaction conditions described by Moreno *et al*. (2011). A serial dilutions factors of 1/10 (*i.e.,* 1X, 0.1X, 0.01X. 0.001X and 0.0001X) was used with genomic DNA purified from leaves and galls. 25 µl PCR reactions were carried out using Dream Taq DNA polymerase reagents (Fermentas Life Sciences, Lithuania), with component concentration as specified by the supplier, PCR quality H_2O (Promega Corporation, Madison, Wisconsin, USA), and primers: UBQ10 Fwd360 (5'-TGCATCTCGTTCTCCGATTG-3') and the reverse UBQ10 (5'-GCGAAGATCAGTCGTTGTTGG-3') (Integrated DNA Technologies, Redwood City, CA, USA). All tests were carried out in triplicates.

Purification of *Agrobacterium rhizogenes* Ri plasmid

The Ri *Agrobacterium rhizogenes* plasmid was used as positive control in RAPDs PCR reactions. Plasmids purified from *Agrobacterium rhizogenes*, line A4, were cultured in YEB 1X media (Piñol *et al.*, 1996) and incubated at room temperature (25 °C) for 3-4 days. Plasmid isolation was carried out as described by Li et al. (1995). Plasmids purified from *A. rhizogenes* line A4 were resuspended in 60 µl of sterile water. Extracted DNA was visualized in a 0.8% agarose gel, showing a high molecular weight and clearly defined fragment.

Purification and re-amplification of previously differentially amplified DNA fragments

Some of the differentially amplified DNA fragments obtained from RAPD assays were isolated and purified to re-amplify them. Purification was performed using the QIAquick Gel Extraction Kit (QIAGEN, Hilden,

Germany) and the Wizard PCR Preps DNA Purification Systems (Promega Corporation, Madison, Wisconsin, USA). These re-amplifications were performed to increase the concentration of purified fragments for cloning and sequencing. To verify the presence of the differentially amplified fragments from gall samples (from RAPD and re-amplification) PCR was performed following the same reaction conditions as above. Negative control for these PCR reactions did not amplify any band.

Cloning of differentially amplified DNA fragments from *Manihot esculenta* galls

Cloning of differentially amplified DNA fragments from *Manihot esculenta* galls were done in *Escherichia coli* XL1-Blue, transfected with pAMP1 cloning vectors (Invitrogen-Life Technologies). Cloning, ligation, transformation, and other DNA manipulations were performed according to standard procedures (Ausubel et al., 1995) or as recommended by the supplier. Fragments of different size and selected for cloning correspond to the ones purified and reamplified from different gall samples. Insertion of the target fragment was corroborated through PCR amplification. PCR reactions were performed using M13 PUC Forward and M13 PUC Reverse as well as SP6 and T7 primers. PCR products were separated and visualized by agarose gel electrophoresis to 1.5 %, TBE 0.5 X, Gel Red 1X, and 75 volts.

Bioinformatic analysis

Overview of Next Generation Sequencing analysis approach

We analyzed the presence of any endophytic microorganisms or possible DNA insertion sequences in the gall tissues comparing samples from healthy plant tissue and gall tissue from casava plants, using a high throughput sequencing platform. The generated data, composed of millions of reads, were analyzed using powerful computational tools. To identify possible exogenous DNA sequences in the gall tissues, a bioinformatic approach, which we named Host Discriminant Genomic Analysis (HDGA), was used. This methodological approach consists of a bioinformatic analysis in which the sequenced data were processed to separate, assemble, and analyze the DNA sequences different from those in the referenced host plant genome. The differing reads resulting from the matches between healthy tissue and gall tissue were separated, thus selecting only the specific reads presented in the DNA from gall tissue (readings having different bases). Sequenced reads between healthy cassava tissue samples, sequenced from genotype Valencia, and the cassava reference genome, CV AM560-2 (Phytozome genome ID: 520 Bredeson et al. 2016) were contrasted and mapping reads to the reference were filtered out. Polymorphic variants reads between the sequenced genomes of healthy plants and galls were filtered out, primarily with the reference cassava genome and then by pairing against themselves. Reads that did not map to the cassava reference represent potential foreign DNA from endophytic organism or foreign inserted DNA into the plant gall cell (see Figure S5 for general pipeline-flow diagram-approach). Differing reads detected only in gall tissue were assembled into contigs.

In addition, seven colony-forming units (CFUs) of possible endophytic bacteria from plant gall tissue and two CFUs from insect larval heads of *Iatrophofia brasiliensis* were isolated and sequenced in an Illumina MiSeq platform.

Supplementary results and discussion

Quantification of PCR inhibition level from genomic DNA purified using the specific endogenous gene UBQ-10 of *Manihot esculenta*

High PCR inhibition from *Manihot esculenta* DNA samples was detected and measured when the housekeeping gene UBQ10 was used, resulting in higher values in several gall samples, until an DNA dilution

factor of 1X10 $^{-2}$ was used (data not shown). DNA extraction was performed to ensure high throughput with the least inhibition possible in subsequent PCR-based tests. Despite that effect, remnant inhibition and DNA degradation in cassava plant tissue was detected using as reference the endogenous ubiquitin gene UBQ-10 of *Manihot esculenta* (results not shown).

Characterizing a potential gall molecular marker

The presence of possible exogenous DNA sequences in gall tissue of *Manihot esculenta* plants was initially determined by the detection of differentially amplified fragments of DNA extracted from these structures by a modified RAPDs technique (Random Amplification Polymorphic DNA). Samples of healthy leaf and gall tissues, growing in the same plant organ, were compared. The aim of this approach was to detect amplification fragments present in the DNA of the gall and not present in the same healthy tissue. We noted the presence of a 550 bp fragment, amplified in many of the gall samples, as well as a clear fragment of approximately 1100 bp and another fragment with an approximated size of 1600 bp. The polymorphism observed when the ipt-Forward and iaaM-Reverse primer were used for RAPDs could indicate the preliminary evidence of foreign genetic material in the plant gall cells.

The consensus DNA sequence obtained from the alignment of RAPDs differentially amplified fragments was used as a template to design and test specific primers as PCR markers for the detection of a putative exogenous DNA or, a DNA insertion sequence present only in gall cells but not in the healthy plant tissues (Supplementary Data 1). PCR reaction was optimized to amplify the expected fragment under high stringency conditions, 6-celsius degrees above annealing temperature (62ºC). Nonetheless, some non-expected amplification bands were occasionally also amplified in leaves and galls of different samples, but the expected band was always present in the gall samples (Figure 1E), which we relate with the amplification of homologous DNA sequences from other endophytic bacteria in the host plant, that could share a similar colonization strategy. This was evidenced by the high and medium identity among DNA fragments amplified and sequenced from wild type plasmids of the putative endophytic bacteria (Figure. 2E), in addition to the amplification of fragments with different sizes than were expected from the same analyzed bacteria plasmids. Nonetheless, determining plasmid relationships is challenging due to the tendency of plasmids to lose, gain, and rearrange genetic content even in the same type of plasmids, therefore, with a tendency to share few phylogenetically concordant core genes (Tazzyman and Bonhoeffer, 2014; Orlek et al., 2017).

Occasionally, some healthy plant tissue samples showed an amplification signal, which we attribute to a possible degradation of the Taq Man probe by still active DNAses detected in DNA purifications (results not shown), which can lead to a false amplification signal. This was confirmed by no DNA amplification products through the PCR reactions using agarose gel electrophoresis and Microchip Electrophoresis System for DNA/RNA (results not shown). Occasional amplification of the expected PCR fragment was also observed in healthy tissue samples in end-point PCR assays, which we attribute to the presence of micro-galls not previously detected in healthy tissue. Furthermore, analysis of one of these unspecific amplified sequenced fragments showed a clear binding site for the forward primer and a less clear binding site for the reverse primer. However, this fragment showed less than 45% of discontinuous identity when compared to the expected sequence (results not shown).

The specific gall fragment (SGF) sequence was analized by BLAST (Basic Local Alignment Search Tool, NCBI) against relevant databases (NCBI GenBank and Integrated Microbial Genomes & Microbiomes (IMG/M) system (https://img.jgi.doe.gov), showing partial pairings with low-medium and often discontinuous length coverage with some ubiquitin-like genes, more specifically, the ubiquitin-like gene E2, a component of the ubiquitin-proteosome system (UPS). Specifically, the ubiquitin-conjugating enzyme (E2) plays a critical role in transporting the ubiquitin-activating enzyme (E1) to the ubiquitin-ligase enzyme (E3), probably

determining whether the labeled protein would be degraded or involved in nonproteolytic processes (Liu et al., 2020). Moreover, some evidence suggests that they could be involved in DNA repair and especially in DNA post-replication repair (Wen et al., 2008; Andersen et al., 2008). However, the role of E2 enzymes in plants remains so far uncharacterized. Otherwise, Blast analysis showed high identity of the cloned RAPD fragment named 4F-2 with the 18S ribosomal RNA gene from several orchid mycorrhizal of the genus *Tulasnellacea*e sp. (Evalue:1.9e-147, Identity: 98% (307/314), Accession: KF266987.1).

Bioinformatic analysis expands the catalog of gall specific sequences and reveal genetic insertion events evidencing plant cell transformation

High throughput sequencing potentially allows full or partial sequencing of any particular genome component in a target sample with enough relative representation. Genome variants between the cassava genotypes sequenced and the reference genome were filtered and separated by comparing the reads of healthy tissues and those from galls. The differing reads resulting from the match between healthy leaf and gall tissues were separated, then selecting only the specific reads presented in the DNA from gall tissue (sequences having different bases when compared to the reference genome). Gall unique reads were assembled into contigs using SPAdes (Prjibelski et al., 2020). The resulting contigs were then mapped against the cassava genome reference to identify hybrid sequences, that is, contigs that contained cassava genome sequences in either the 5', 3' or both ends of the sequence. There were 216 (32%) hybrids fragments harboring sequences with interspersed homology to the host plant genome, which we associate once again with structural DNA rearrangements generated as a consequence of insertion events (Supplementary Data 3). Following this approach, reads that did not map to the cassava reference nor shared between healthy and gall tissue, represent potential foreign DNA from endophytic organisms, such as bacteria or fungi, or possible foreign DNA integrated into the genome of gall cells (see Figure S3 for general pipeline-flow diagram approach, Figure 3-A and Supplementary Data 3). Some of these endophytic organisms may be involved in gall induction and formation.

Metagenomes reveal an enriched microbial community in galls

Functional characteristics attributed to some of the exclusive or enriched gall microbiome components include the production of vitamin B12 (Fang et al. (2017), and the conversion of nitrate (NO3) into nitrogenous compounds (N2) by *Pseudomonas denitrificans*, while the plant growth-promoting bacteria *Pseudomonas nitroreducens* improves cell development and enhance nitrate uptake in plants (Trinh et al., 2018). Potassium-solubilizing bacteria (KSB) *Cupriavidus axalaticus* could play a fundamental role in solubilizing fixed potassium and consequently making this essential element accessible to plants. *Cupriavidus axalaticus* T2 strain of this bacterium has been related to the simultaneous degradation capability of phenolic compounds and denitrification under aerobic conditions to produce ammonium as well (Yan et al., 2021).

Meanwhile, shared species between gall and healthy tissue of the genus *Hydrogenophaga* (Gan et al., 2011), as well as *Pseudomonas* bacterial strains have been related to biodegradation of the potentially harmful compounds 4-Aminobenzenesulfonate, resulting in the release of ammonium and sulfate. Transformation and fixation of nitrogen compounds have been associated also with *Acinetobacter baumannii* along with the synthesis of IAA (Lin et al., 2018), and even this endophyte can help hosts to remove pollutants and withstand environmental stress (Khaksar et al., 2017). Endophytic species of *Halomonas* and *Pseudomonas* (detected only in gall tissue) have been associated with alleviation of the toxic effects of salinity (Zinniel et al., 2002; Zhang et al., 2020). *Guillardia theta* and *Stanieria cyanosphaera* cyanobacteria could simultaneously increase the photosynthesis carried out in the gall tissue (Figure 3B).

Achromobacter deleyi also appears to be an exclusive gall bacterium using a filter resolution of 5K, considered a significant but conservative value in the analysis (results not shown). This potentially exclusive gall

bacterium was reported as a component of the microbiome in legume nodules in *Mimosa pudica* (Tapia-García, 2020). Likewise, *Phytobacter ursingii* for instance, has potentially been associated with nitrogen-fixation, despite no environmental isolates of this species being reported so far (Pillonetto, et al. 2017). Likewise, *Pseudomonas putida* produces and degrades IAA (Leveau and Gerards, 2008), and *Achromobacter xylosoxidans* is reported to have plant growth-promoting activity of IAA (Jha and Kumar, 2009). Moreover, this approach allows us to taxonomically classify read sequences, revealing a possible minor and exclusive community component of gall microbiome as well.

Analysis of taxa associated with exclusive gall reads did not map to the reference cassava genome was attempted. A significant amount of exclusive gall reads were associated with several of the same *Pseudomonas* bacterial species reported as exclusive or enriched in the gall tissue (Figure 3C, 3D). According to this result, we hypothesize that different regions of the bacterial genome could have been differentially sequenced between samples, but with sufficient relative abundance to be reflected in this taxonomic profile.

Moreover, the taxonomic profile carried out with the samples of healthy leaf tissue sequenced, and functional characteristics reported in the literature associated with some of these microorganisms, showed that some could be not only beneficial to the cassava plant, but may also include opportunistic pathogens in the microbiome (Figure 3D).

Particular functional analysis (GOs) of gall endophytes and putative endosymbionts of the inducing insect

Significant particular GOs of endophytic bacteria and from possible insect endosymbiotic bacteria are shown in Supplementary Data 6 as well. Among the specific functions and biological processes inferred from endophytic bacteria, the most intriguing are those related to pathogenesis (GO:0009405, value:1.3e-08), cellular amino acid biosynthetic process (GO:0008652, value:5,00E-06), biological process involved in symbiotic interaction (GO:0044403, value:5.1e-06), response to host immune response (GO:0052572, 6.6e-05), sulfur compound biosynthetic process (GO:0044272, value:0.00017), urea cycle (GO:0000050, velue:0.00018), response to nitrosative stress (GO:0051409, value:0.00044), regulation of immune response (GO:0050776, velue:0,0008), recombinational repair (GO: GO:0000725, value:0.00101), modulation by symbiont of host immune response (GO:0052553, value:0.00108), chromosome organization (GO:0051276, value:0.00128), double-strand break repair (GO:0006302, value:0.00202), positive regulation of DNA binding (GO:0043388, value:0.00215), organonitrogen compound biosynthetic process (GO:1901566, value:0.00316), regulation of DNA metabolic process (GO:0051052, value:0.0034), regulation of meiosis I (GO:0060631, value:0.0036), mitigation of host defenses by symbiont (GO:0030682, value:0.00362), chaperone-mediated protein folding (GO:0061077, value:0.00501), regulation of DNA-dependent DNA replication (GO:0090329, value:0.00643). Likewise, some relevant GOs associated with the putative endosymbiont genomes, include those related with drug metabolic process (GO:0017144, value:2.4e-06), cellular amino acid biosynthetic process (GO:0008652, value: 6.2e-06), urea cycle (GO:0000050, value:0.00011), organonitrogen compound biosynthetic process (GO:1901566, value:0.00065), response to host immune response (GO:0052572, value:0.00228), regulation of meiosis I (GO:0060631, value:0.00233), import into cell (GO:0098657, value: 0.00235), antibiotic catabolic process (GO:0017001, value:0.00294), regulation of DNA-dependent DNA replication (GO:0090329, value:0.00317), regulation of neurotransmitter levels (GO:0001505, value: 0.00328), double-strand break repair via homologous recombination (GO:0000724, value:0.00568), regulation of chromosome organization (GO:0033044, value:0.00634), positive regulation of nitrogen compound metabolic process (GO:0051173,value:0.00662), and DNA conformation change (GO:0071103, value:0.00662), among many others with significant value (Supplementary Data 6).

Moreover, direct wild type plasmid sequencing was attempted by purifying enrichment bacterial plasmid DNA using standard sequencing techniques from Illumina MiSeq sequencing platform. However, presumably due to the stability and integrity of plasmid DNA during the fragmentation procedure, no sequencing was done for any endosymbiotic and endophytic bacteria wild type plasmid.

Scanning electron microscopy (SEM) and Transmission electron microscopy (TEM) of gall tissues

Structures shown in Figure S7 by Scanning Electron Microscopy (SEM) and Transmission Electron Microscopy (TEM), are consistent in morphology and size with intracellular bacterial cells (Lebsky and Poghosyan, 2014; Mensi et al., 2014; Zhou et al., 2014; War and Joshi, 2017; Esposito-Polesi et al, 2017). Moreover, some gall cells reveal bacteria added or embedded in a possible type of biofilm (Figures S7C, E), like those described by Esposito-Polesi et al. (2017) and Taufiq and Darah (2020). These possible endophytic microorganisms can also be observed along with possible plastids like amyloplasts of different sizes (Lebsky and Poghosyan, 2014; Gama et al., 2015; Olguin-Maciel et al., 2017) (Figures S7D, E). At least nine possible morphospecies of bacteria were observed in TEM images obtained from cassava gall tissues, some of them are shown in the Figures S7F-J. Although an exhaustive comparative search with respect to normal leaf tissue was not performed, the abundance of endophytic microorganisms seems to be greater in the gall tissues in relation to the surrounding leaf tissues (images not shown), which is consistent with the results of genomic analyses (Host Discriminant Genomic Analysis, HDGA).

SI References

Andersen, P. L., Xu, F., and Xiao, W. (2008). Eukaryotic DNA damage tolerance and translesion synthesis through covalent modifications of PCNA. *Cell Res.*, 18, 162–173.

Ausubel, M., Brent, R., Kingston, R.E., Moore, D.D., Seidman, J.G., Smith, J.A., and Struhl, K. (1995). Current Protocols in Molecular Biology. John Wiley & Sons, Inc., New York, USA. doi: 10.1002/mrd.1080010210

Barash, I., and Manulis-Sasson, S. (2009) Recent evolution of bacte- rial pathogens: the gall forming Pantoea agglomerans case. Annu. Rev. *Phytopathol.* 47, 133–152.

Borriello, G., Russo, V., Paradiso, R., Riccardi, M. G., Criscuolo, D., Verde, G., Marasco, R., Pedone, P. V., Galiero, G., and Baglivo, I. (2020). Different impacts of MucR Binding to the babR and virB Promoters on gene expression in *Brucella abortus* 2308. *Biomolecules* 10 (5), 788. doi: 10.3390/biom10050788

Bredeson, J. V., Lyons, J. B., Prochnik, S. E., Wu, G. A., Ha, C. M., Edsinger-Gonzales, E., et al. (2016). Sequencing wild and cultivated cassava and related species reveals extensive interspecific hybridization and genetic diversity. *Nat Biotechnol.* 34 (5), 562–570. doi: 10.1038/nbt.3535

Bushnell, B. (2015). BBMap short read aligner, and other bioinformatic tools.

Esposito-Polesi, N. P., de Abreu-Tarazi, M. F., de Almeida, C. V., Tsai, S. M., and de Almeida, M. (2017). Investigation of endophytic bacterial community in supposedly axenic cultures of pineapple and orchids with evidence on abundant intracellular bacteria. Current Microbiology, 74 (1), 103–113. doi: 10.1007/s00284-016-1163-0

Fang, H., Kang, J. and Zhang, D. (2017). Microbial production of vitamin B12: a review and future perspectives. Microb Cell Fact 16, 15. doi: 10.1186/s12934-017-0631-y

Gama, T., Lucas, F., Macedo, E. (2015). Morphology of starch grains of cassava cultivars (*Manihot esculenta*

Crantz, Euphorbiaceae) –Pará, Brazil. *Acta Biológica Catarinense* 2, 10.21726/abc.v2i1.194.

Hall, T. A. (1999). BioEdit: A user-friendly biological sequence alignment editor and analysis program for Windows 95/98/NT. *Nucleic Acids Symp. Ser*. 41, 95–98.

Jha, P., Kumar, A. (2009). Characterization of novel plant growth promoting endophytic bacterium *Achromobacter xylosoxidans* from wheat plant. *Microb. Ecol.* 58, 179–188. https://doi.org/10.1007/s00248-009-9485-0

Khaksar, G., Treesubsuntorn, C., and Thiravetyan, P. (2017). Impact of endophytic colonization patterns on *Zamioculcas zamiifolia* stress response and in regulating ROS, tryptophan and IAA levels underairborne formaldehyde and formaldehyde-contaminated soil conditions. *Plant Physiol. Biochem*. 114, 1–9.

Lebsky, V., Poghosyan, A. (2014). Scanning electron microscopy detection of phytoplasmas and other phloem limiting pathogens associated with emerging diseases of plants. In A. Méndez-Vilas (Eds.). Microscopy: advances in scientific research and education. Formatex Research Center Publisher. Vol. 1 (pp. 1-588).

Leveau, J. H., and Gerards, S. (2008). Discovery of a bacterial gene cluster for catabolism of the plant hormone indole 3-acetic acid. *FEMS Microbiol. Ecol.* 65, 238–250.

Li, X. Q., Stahl, R., and Brown, G. (1995). Rapid micropreps and minipreps of Ti plasmid and binary vector from *Agrobacterium tumefaciens*. *Transgenic Research* 4, 349- 351.

Lichter, A., Barash, I., Valinsky, L., and Manulis, S. (1995) The genes involved in cytokinin biosynthesis in *Erwinia herbicola* pv. *gypsophilae*: characterization and role in gall formation. J Bacteriol. 177, 4457–4465. doi: 10.1128/jb.177.15.4457-4465.1995

Lin, H. R., Shu, H. Y., and Lin, G. H. (2018). Biological roles of indole-3-acetic acid in *Acinetobacter baumannii*. *Microbiological Research* 216. doi: 10.1016/j.micres.2018.08.004.

Liu, W., Tang, X., Qi, X., Fu, X., Ghimire, S., Ma, R., Li, S., et al. (2020). The ubiquitin conjugating enzyme: An important ubiquitin transfer platform in ubiquitin-proteasome system. *International Journal of Molecular Sciences* 21 (8), 2894. MDPI AG. Retrieved from http://dx.doi.org/10.3390/ijms21082894

MacDonald, E. M. S., Powell, G. K., Regier, D. A., Glass, N. L., Roberto, F., Kosuge, T., and Morris, R. O. (1986) Secretion of zeatin, ribosylzeatin, and ribosyl-1"-methylzeatin by *Pseudomonas savastanoi*. *Plant Physiol* 82, 742–747. doi:10.1104/pp.82.3.742

Mensi, I., Vernerey, M. S., Gargani, D., Nicole, M., and Rott, P. (2014). Breaking dogmas: the plant vascular pathogen *Xanthomonas albilineans* is able to invade non-vascular tissues despite its reduced genome. *Open Biology* 4 (2), 130116. doi: 10.1098/rsob.130116

Nissan, G., Chalupowicz, L., Sessa, G., Manulis-Sasson, and S., Barash, I. (2019), Two *Pantoea agglomerans* type III effectors can transform nonpathogenic and phytopathogenic bacteria into host-specific gall-forming pathogens. *Molecular Plant Pathology* 20, 1582-1587. doi: 10.1111/mpp.12860

Moreno, I., Gruissem, W., and Vanderschuren, H. (2011). Reference genes for reliable potyvirus quantitation in cassava and analysis of Cassava brown streak virus load in host varieties. *Journal of Virology Methods* 177, 49-54.

Olguin-Maciel, E., Larqué-Saavedra, A., Pérez-Brito, D., Barahona-Pérez, L., Alzate-Gaviria, L., Toledano-Thompson, T., et al. (2017). *Brosimum alicastrum* as a novel starch source for bioethanol production. *Energies* 10 (10), 1574. MDPI AG. Retrieved from doi: 10.3390/en10101574

Orlek, A., Stoesser, N., Anjum, M. F., Doumith, M., Ellington, M. J., Peto, T., Crook, D., Woodford, N., Walker, A. S., Phan, H., and Sheppard, A. E. (2017). Plasmid classification in an era of whole-genome sequencing: Application in studies of antibiotic resistance epidemiology. *Frontiers in Microbiology* 8, 182. doi: 10.3389/fmicb.2017.00182

Pillonetto, M., Arend, L. N., Faoro, H., D'Espindula, H., Blom, J., Smits, T., Mira, M. T., and Rezzonico, F. (2018). Emended description of the genus Phytobacter, its type species *Phytobacter diazotrophicus* (Zhang 2008) and description of *Phytobacter ursingii* sp. nov. *International Journal of Systematic and Evolutionary Microbiology* 68 (1), 176–184. doi: 10.1099/ijsem.0.002477

Prjibelski, A, Antipov, D., Meleshko, D., Lapidus, A., and Korobeynikov, A. (2020). Using SPAdes De Novo Assembler. *Curr. Protoc. Bioinformatics* 70 (1), e102. doi: 10.1002/cpbi.102. doi: 10.1002/cpbi.102

Stokes, H. W., and Gillings, M. R. (2011). Gene flow, mobile genetic elements and the recruitment of antibiotic resistance genes into Gram-negative pathogens. *FEMS Microbiol. Rev.* 35, 790–819. Doi: 10.1111/j.1574-6976.2011. 00273.x

Tapia-García, E. Y., Hernández-Trejo, V., Guevara-Luna, J., Rojas-Rojas, F. U., Arroyo-Herrera, I., Meza-Radilla, G., Vásquez-Murrieta, M.S., and Estrada-de los Santos, P. (2020). Plant growth-promoting bacteria isolated from wild legume nodules and nodules of *Phaseolus vulgaris* L. trap plants in central and southern Mexico. *Microbiological Research* 239, 126522.

Taufiq, M. M. J., and Darah, I. (2020). Antibacterial and antibiofilm activities of crude extract of *Lasiodiplodia pseudotheobromae* IBRL OS-64 against foodborne bacterium, *Yersinia enterocolitica*. Journal of Pharmaceutical Research International, 32 (14), 87-102. doi: 10.9734/jpri/2020/v32i1430609

Tazzyman, S. J., and Bonhoeffer, S. (2014). Why there are no essential genes on plasmids. Mol. Biol. Evol. 32, 3079–3088.

Trinh, C. S., Lee, H., Lee, W. J., Lee, S. J., Chung, N., Han, J., Kim, J., Hong, S. W., and Lee, H. (2018). Evaluation of the plant growth-promoting activity of *Pseudomonas nitroreducens* in *Arabidopsis thaliana* and *Lactuca sativa*. *Plant Cell Reports* 37 (6), 873–885. doi: 10.1007/s00299-018-2275-8

War, Nongkhlaw, F. M. Joshi, and S. R. (2017). Microscopic study on colonization and antimicrobial property of endophytic bacteria associated with ethnomedicinal plants of Meghalaya. J*ournal of Microscopy and Ultrastructure* 5 (3), 132–139. doi: 10.1016/j.jmau.2016.09.002

Wen, R., Torres Acosta, J. A., Pastushok, L., Lai, X., Pelzer, L., Wang, H., and Xiao, W. (2008). *Arabidopsis* UEV1D promotes Lysine-63-linked polyubiquitination and is involved in DNA damage response. *Plant Cell* 20, 213.

Yan, S., H., Kuang, X., Luo, L., Zhou, ., and Zhou, . (2021). Characteristics and mechanism of simultaneous nitrate and phenol removal by a newly isolated *Cupriavidus oxalaticus* T2. *International Biodeterioration & Biodegradation* 161, 105234. doi: 10.1016/j.ibiod.2021.105234.

Yang, X. M., Hui, Y., Zhao, L. Q., Zhu, D. H., Zeng, Y., and Yang, X. H. (2021). Comparison of auxin and cytokinins concentrations, and the structure of bacterial community between host twigs and *Lithosaphonecrus arcoverticus* galls. *Insects* 12 (11), 982. doi: 10.3390/insects12110982

Yang, X., Hui, Y., Zhu, D., Zeng, Y., Zhao, L., Yang, X., and Wang, Y. (2022). The diversity of bacteria associated with the invasive gall wasp *Dryocosmus kuriphilus*, its galls and a specialist parasitoid on chestnuts. *Insects* 13 (1), 86. doi:10.3390/insects13010086

Zhang, J., Wang, P., Tian, H., Tao, Z., and Guo, T. (2020). Transcriptome analysis of ice plant growth-promoting endophytic bacterium *Halomonas* sp. strain MC1 to identify the genes involved in salt

tolerance. *Microorganisms* 8 (1), 88. doi: 10.3390/microorganisms8010088

Zinniel, D. K., Lambrecht, P., Harris, N. B., Feng, Z., Kuczmarski, D., Higley, P., Ishimaru, C. A., Arunakumari, A., Barletta, R. G., and Vidaver, A. K. (2002). Isolation and characterization of endophytic colonizing bacteria from agronomic crops and prairie plants. *Appl. Environ. Microbiol.* 68, 2198.

Zhou, J., Zhao, X., and Dai, C. (2014), Antagonistic mechanisms of endophytic *Pseudomonas fluorescens* against *Athelia rolfsii*. *J. Appl. Microbiol.* 117, 1144-1158. doi: 10.1111/jam.12586

SUPLEMENTARY FIGURES

Figure S1. Inducing insect, *Iatrophobia brasiliensis*. (A) Larva. **(B)** Adult. (**C-D)** Salivary glands dissected from larva.

Figure S2. *Rhodococcus* bacterial strain isolated from the gall-inducing insect (larval head) associated with gall induction in *Manihot esculenta*. **(A)** Culture on YEB 1X media at 26 °C. **(B)** Cultured and storaged after more than one month refrigerated at 4-10 celsius degrees, showing pinksh color. **(C, D)** Gram-positive bacillus shown in light microscope.

Figure S3. Randomly Amplified Polymorphic DNA (RAPD) specific to gall samples. **(A)** Theoretical diagram for detection of differentially amplified fragments showing the hypothetical binding sites of primers and the respective amplified bands from healthy plant tissue and from gall tissue. Case1: differentially amplified band with one primer binding to plant DNA and the other binding to the inserted DNA sequence. Case 2: differentially amplified band with both primers binding sites to the inserted DNA sequence. Case 3: differentially amplified band with both primers binding to the genome of a potentially endophytic microorganism. **(B)** Agarose gel electrophoresis of amplified DNA fragments ranging from 100 to 4500 base pairs, comparing healthy leaf tissue DNA samples (H) and gall tissue DNA samples (G). M: Lamda DNA EcoRI/Hind-III Marker; NC: negative control (reagents only), lines S1-S8: samples of healthy leaf and gall tissues growing in the same plant (pair-compared). Arrows point to differentially amplified fragments in paired samples. PC: “positive” sample from *Agrobacterium rhizogenes* plasmids. **(C)** Analytical detections of the RAPDs amplicons through Microchip Electrophoresis System for DNA/RNA (MultiNA) show the amplification of differentially amplified fragments. Healthy leaf tissue DNA samples (H) and gall tissue DNA samples (G) pair-compared; MM: molecular markers ΦX174 DNA/Hae III marker (Promega) and 25 bp DNA marker (Invitrogen), NC: negative control (reagents only). Red arrows point to differentially amplified fragments in paired samples.

Figure S4. Gel electrophoresis of PCR amplicons using primers specific for the specific gall fragment marker (SGF). Samples correspond to purified wild type plasmids from two putative endosymbiotic bacteria of the genus *Rhodococcus* and *Pseudomonas,* isolated from the larval head of the inducing insect *Iatrophofia brasiliensis* (colony-forming units ISB 1 and ISB 2), as well as wild type plasmids purified from seven endophytic bacteria isolated from cassava gall tissue (IEB). PCR amplified fragments are also shown for the inducing insect salivary gland sample (SG). Samples of DNA purified from healthy leaf and gall tissues were used as positive controls (lines S1-S4). Green circles indicate positive amplification for SGF (S1-S3). M: molecular weight marker (Gene Ruler 1 KB Plus), NC: negative control (reagents only).

Figure S5. Host Discriminant Genomic Analysis (HDGA) workflow. High throughput sequencing data from samples of leaf healthy tissue and insect-induced galls were quality controlled (step 1) and compared against

the cassava reference genome to separate reads with near perfect match sequences (step 2). The differing *reads* between healthy tissue and gall tissue were filtered-separated, thus selecting reads unique to DNA from gall tissue (step 3). Reads that did not map to the cassava reference represent potential foreign DNA from endophytic organism or foreign DNA inserted into the gall cell. Differing reads detected only in DNA from gall tissue were assembled into contigs (step 4). Contigs were then mapped to the cassava reference again (step 5) and the resulting alignments filtered (step 6), leaving only high-quality alignment of hybrid contigs: containing a fragment of both host DNA and foreign (possibly) inserted DNA. Annotation of the filtered alignments was done with MegaBlast, which revealed known candidate genes associated with the inserted sequences (step 7). Finally, taxonomic profiles of the gall unique contigs was conducted and the internal gall microbiome was determined (step 8).

Figure S6. Endophytic bacteria determined by synteny analysis. **(A-C)** Colony-forming units (CFUs) of potentially endophytic bacteria and one fungal growth isolated from surface-sterilized *Manihot esculenta* gall slices. Each of these CFUs were named IEB (isolates of endophytic bacteria). **(D-F)** Sequential isolation of CFUs from initial growths (2-3 sequential isolates). **(G-M)** Synteny analysis using specific galls contigs as target sequences versus each of the sequenced bacterial genomes.

Figure S7. Scanning Electron Microscopy (SEM) and Transmission Electron Microscopy (TEM) images of possible endophytes microorganisms in gall tissue in *Manihot esculenta*. **(A)** SEM of a cross section of cassava gall from the internal cavity (IC) to the outer epidermis. IZ: internal zone, MZ: middle zone, EZ: external zone. **(B-E)** SEM images showing possible intracellular bacteria cells. **(F-J)** TEM images showing possible intracellular bacteria cells. Intracellular microorganisms are pointed out with white arrows. Fungal hyphae or filamentous bacterium are pointed out with grey arrows, c. Possible biofilms are marked with a black arrow. st: starch granules.

Supplementary Data

Supplementary Data 1: Alignment of RAPDs differentially amplified fragments of Specific Gall Fragment.

Supplementary Data 2: All Specific Gall Contigs sequences.

Supplementary Data 3: Selected Specific Gall Hybrid Contigs sequence information.

Supplementary Data 4: Blast Analysis and Annotation of Specific Gall Hybrid Contigs.

Supplementary Data 5: MegaBlast and Annotation of All Specific Gall Contigs.

Supplementary Data 6: Significant discrete GO terms from endophytic bacteria and possible insect endosymbiotic bacteria.
