# Supplementary material for: Microbiome and plant cell transformation trigger insect gall induction in cassava
Source: Front Plant Sci. 2023 Nov 29;14:1237966. doi: 10.3389/fpls.2023.1237966 (PMC10731979; doi:10.3389/fpls.2023.1237966)
Supplement: Supplementary file 1 [file DataSheet_1.zip › Supplementary Material/Supplementary Data_1_Alignment of RAPDs differentially amplified fragments of Specific Gall Fragment.pdf]

Alignment: A:\Sequence alignment 1,2,3.txt

Primer Forward

```
             ....|....| ....|....| ....|....| ....|....| ....|....| ....|....|
                 5         15         25         35         45         55
OG. 1  A81   ---GAGGCCC TTGACATGN- TCTGGAGCGG AGCANNTCTA CAATGATGNC CAAGGCCG-A
OG. 2  A10   ----GAGNCC TTGACATGN- TCTGGAGCGG AGCANTTCTA CAATGATGTC CNAGGCCCGA
OG. 3  A11   GNNNGAGNCC TTGACATGNT TNTGGAGCGG AGCCCNTCTA CAATGATGTC CNAGGCCG-A
Clustal Co         * ** *********  * ******** ***   **** ******** * * *****  *

             ....|....| ....|....| ....|....| ....|....| ....|....| ....|....|
                  65         75         85         95        105        115
OG. 1  A81   CCTCACGATG ATCTTCGCTT NNNAAAGNAT AGGCGCATCG GCNAGGACGG CGATCACGGC
OG. 2  A10   CCTCACGATG ATCTTCGCTT NNAAAAGTAT AGGCGCATCG GCAAGGACGG CGATCACGGC
OG. 3  A11   CCTCACGATG ATCTTCGCTT TTAAAAGTAT AGGCGCATCG GCAAGGACGG CGATNNNGGC
Clustal Co   ********** **********    **** ** ********** ** ******* ****   ***

             ....|....| ....|....| ....|....| ....|....| ....|....| ....|....|
                125        135        145        155        165        175
OG. 1  A81   GCATTTCCTG ACCTGGTGAC GGCTGATCAT CTGACATCCA CNAGATCCGN CAGCAAGTCC
OG. 2  A10   GCATTTCCTG ACCTGGTGAC GGCTGATCAT CTGACATCCA CGAGATCCGT CAGCAAGTCC
OG. 3  A11   GCATTTCCTG ACCTGGTGAC GGCTGATCAT CTGACATCCA CGAGATCCGT CAGCAAGTCC
Clustal Co   ********** ********** ********** ********** * *******  **********

             ....|....| ....|....| ....|....| ....|....| ....|....| ....|....|
                185        195        205        215        225        235
OG. 1  A81   GTGTCATTCC CACTCGTCGC CATGCAATTC ACCCTGCGCC ACTTTGTTCG CTGCACAGAG
OG. 2  A10   GTGTCATTCC CACTCGTCGC CATGCAATTC ACCCTGCGCC ACTTTGTTCG CTGCACAGAG
```

```
OG. 3   A11    GTGTCATTCC CACTCGTCGC CATGCAATTC ACCCTGCGCC ACTTTGTTCG CTGCACAGAG
Clustal Co     ********** ********** ********** ********** ********** **********

               ....|....| ....|....| ....|....| ....|....| ....|....| ....|....|
                   245        255        265        275        285        295
OG. 1   A81    TTCTGTCTGA TCTGCCACCG GACCCTACCG AGCGAAATCG AAGCACTCAA GCCTTACGTC
OG. 2   A10    TTCTGTCTGA TCTGCCACCG GACCCTACCG AGCGAAATCG AAGCACTCAA GCCTTACGTC
OG. 3   A11    TTCTGTCTGA TCTGCCACCG GACCCTACCG AGCGAAATCG AAGCACTCAA GCCTTACGTC
Clustal Co     ********** ********** ********** ********** ********** **********

               ....|....| ....|....| ....|....| ....|....| ....|....| ....|....|
                   305        315        325        335        345        355
OG. 1   A81    TGCGACAACC CACTGTGCCT TTACCAGTAC ATGAGTCTCG GTTTCGGTCC AAGCATTGAT
OG. 2   A10    TGCGACAACC CACTGTGCCT TTACCAGTAC ATGAGTCTCG GTTTCGGTCC AAGCATTGAT
OG. 3   A11    TGCGACAACC CACTGTGCCT TTACCAGTAC ATGAGTCTCG GTTTCGGTCC AAGCATTGAT
Clustal Co     ********** ********** ********** ********** ********** **********

               ....|....| ....|....| ....|....| ....|....| ....|....| ....|....|
                   365        375        385        395        405        415
OG. 1   A81    CATGAGATCA TCACTCAACC ACTGGTCATC GATCTGCTCA TCTCATTCTG CTACAGCAGT
OG. 2   A10    CATGAGATCA TCACTCAACC ACTGGTCATC GATCTGCTCA TCTCATTCTG CTACAGCAGT
OG. 3   A11    CATGAGATCA TCACTCAACC ACTGGTCATC GATCTGCTCA TCTCATTCTG CTACAGCAGT
Clustal Co     ********** ********** ********** ********** ********** **********
                                                                 Primer Reverse
               ....|....| ....|....| ....|....| ....|....| ....|....| ....|....|
                   425        435        445        455        465        475
OG. 1   A81    GCTGCGAATG GCATTCTCAA GGACTTTCCT GTGGGATTGG CGATCACAGT ACCACGCTCG
OG. 2   A10    GCTGCGAATG GCATTCTCAA GGACTTTCCT GTGGGATTGG CGATCACAGT ACCACGCTCG
OG. 3   A11    GCTGCGAATG GCATTCTCAA GGACTTTCCT GTGGGATTGG CGATCACAGT ACCACGCTCG
Clustal Co     ********** ********** ********** ********** ********** **********
```

```
              ....|....| ....|....| ....|....| ....|.
                 485        495        505        515
OG. 1  A81    TTCACCGGGN NNN--NCNNG NNN------- ------
OG. 2  A10    TTCACCGAGN NNNG-CCTTG CNAAANTNAT GANCTN
OG. 3  A11    TTCACCGGAA NNN---CCTG NCAAACTNAT NANCN-
Clustal Co    *******    ***   *  *
```
