# Supplementary material for: Microbiome and plant cell transformation trigger insect gall induction in cassava
Source: Front Plant Sci. 2023 Nov 29;14:1237966. doi: 10.3389/fpls.2023.1237966 (PMC10731979; doi:10.3389/fpls.2023.1237966)
Supplement: Supplementary file 1 [file DataSheet_1.zip › Supplementary Material/Script_for_Host_Discriminant_Genomic_Analysis.pdf]

```bash
#!/bin/bash

# Host Discriminant Genomic Analysis

######################################################################
#########
# Sample data: Bioproject PRJNA905450
# Reference genome: Bioproject PRJNA234389
######################################################################
#########

# STEP 1: Quality control
for file in ./*.fastq.gz; do fastqc $file; done
for file in ./*.fastq.gz; do name=$(basename $file .fastq.gz);
trimmomatic PE -threads 24  $file ${name}_R2.fastq.gz ${name}
_R1_paired.fastq.gz ${name}_R1_unpaired.fastq.gz ${name}
_R2_paired.fastq.gz ${name}_R2_unpaired.fastq.gz ILLUMINACLIP:TruSeq3-
PE.fa:2:30:10:2:True TRAILING:20 AVGQUAL:20 MINLEN:130

# STEP 2: Align reads to reference
bbmap ref=Mesculenta_520_v7.fasta
for file in ./*_paired.fastq.gz; do name=$(basename $file
_paired.fastq.gz); bbmap -Xmx200g in=$file outm=${name}_map.sam outu=$
{name}_unmap.fastq unpigz=t maxindel=80 strictmaxindel=t
semiperfectmode=t trimq=15 mintrimlength=80 subfilter=20 threads=24;
done
cat 8h_unmap.fastq >> unmap_leaf.fastq && cat 15h_unmap.fastq >>
unmapped_leaf.fastq
cat 13a_unmap.fastq >> unmap_gall.fastq && cat 15a_unmap.fastq >>
unmapped_gall.fastq

# STEP 3: Contrast leaf and gall unmapped reads
bioawk -c fastx '{print ">"$name"\n"$seq}' unmapped_leaf.fastq >
unmapped_leaf.fasta
bbduk -Xmx100g in=unmapped_gall.fastq.gz ref=unmapped_leaf.fasta
out=unmatch_gall.fastq outm=match_gall_leaf.fastq threads=24 qtrim=r
trimq=15 minlength=50 prealloc=t

# STEP 4: Assemble contigs
spades.py -o gall_unique_contigs -s unmatch_gall.fastq --only-
assembler --careful -t 24 -m 200

# STEP 5: Align contigs to reference
bwa index -p yuca Mesculenta_520_v7.fasta
bwa mem -t 24 yuca gall_unique_contigs.fasta >
gall_unique_to_yuca_ref.sam

# STEP 6: Filter alignments
samtools view -@ 24 -q 20 gall_unique_to_yuca_ref.sam >
only_mapped_contigs.sam
```

```
seqtk subseq gall_unique_contigs.fasta only_mapped_contigs.list >
gall_unique_contigs_mapped_to_ref.fasta
bbmap ref=gall_unique_contigs_mapped_to_ref.fasta
bbmap -Xmx100g in=unmatch_gall.fastq outm=unmatch_gall_map.sam
outu=unmatch_gall_unmap.fastq unpigz=t threads=24
scafstats=scafstats.txt
samtools view unmatch_gall_map.sam | cut -f3 >
contigs_with_reads_mapping_to_it.txt
sort contigs_with_reads_mapping_to_it.txt | uniq -c | sort -nr >
reads_per_contigs.txt
```
