# Supplementary figures and images for: Microbiome and plant cell transformation trigger insect gall induction in cassava
Source: Front Plant Sci. 2023 Nov 29;14:1237966. doi: 10.3389/fpls.2023.1237966 (PMC10731979; doi:10.3389/fpls.2023.1237966)

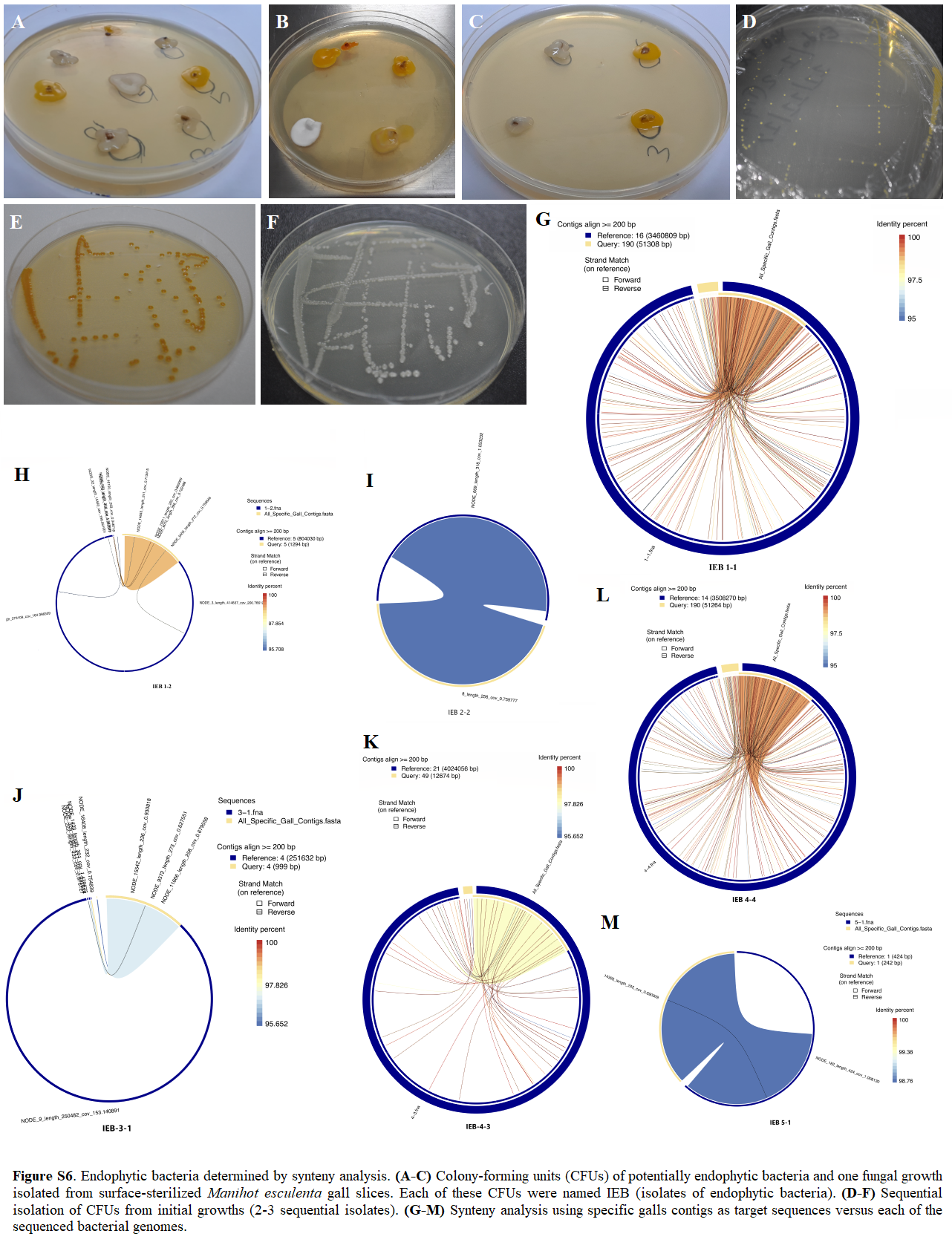

Supplement: Supplementary file 1 [file DataSheet_1.zip › Supplementary Material/Figure_S6_Endophytic bacteria determined by synteny analysis.tif]

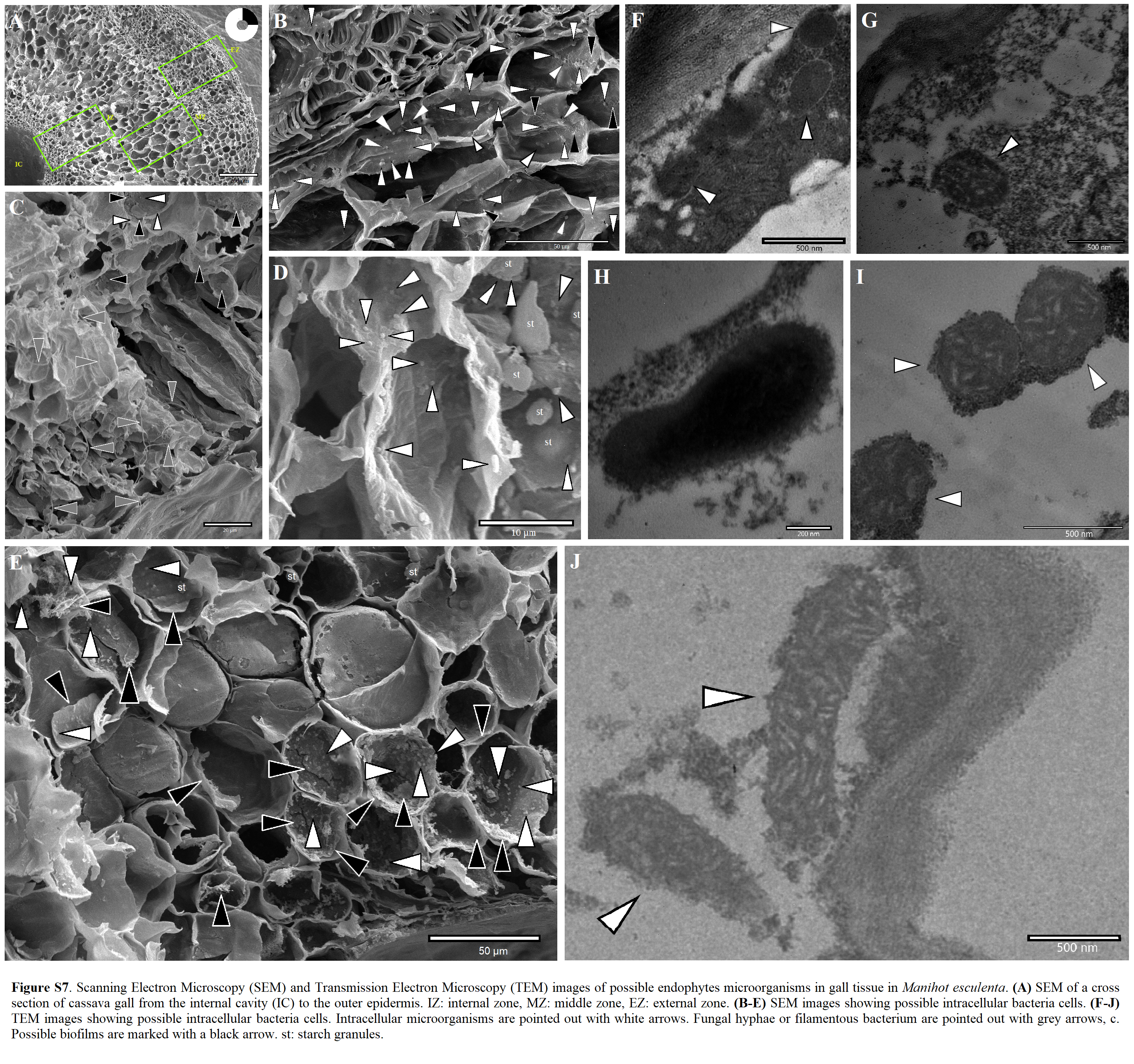

Supplement: Supplementary file 1 [file DataSheet_1.zip › Supplementary Material/Figure_S7_Scanning Electron Microscopy (SEM) and Transmission Electron Microscopy (TEM) images of possible endophytes microorganisms in gall tissue in Manihot esculenta.tif]

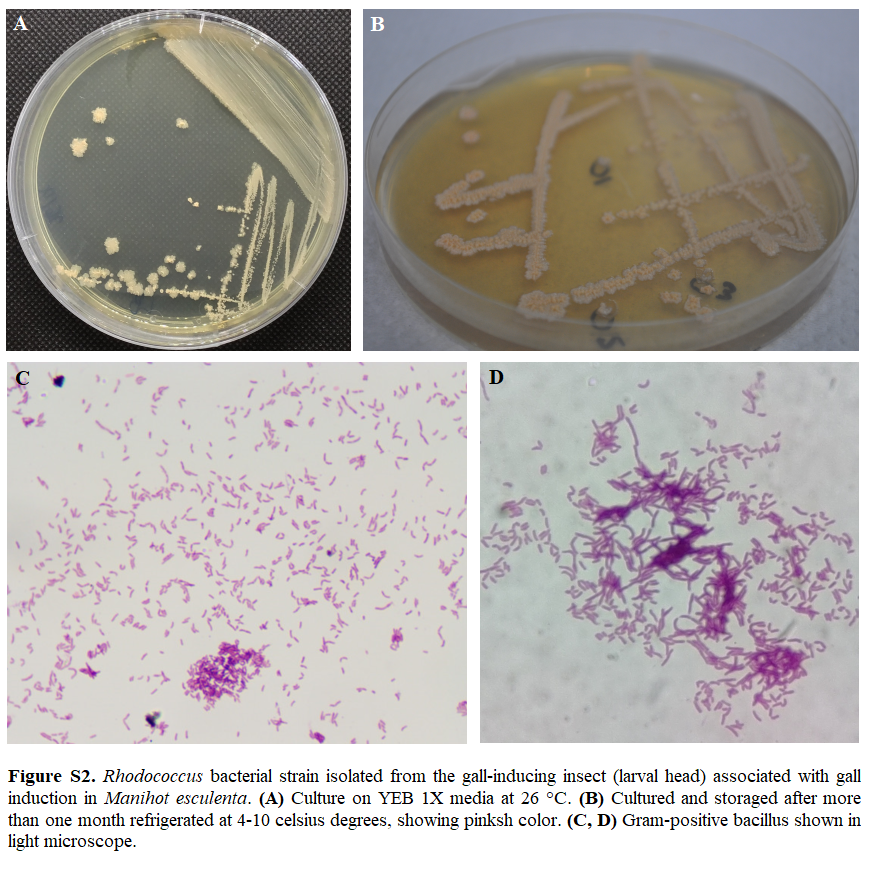

Supplement: Supplementary file 1 [file DataSheet_1.zip › Supplementary Material/Figure_S2_Rhodococcus bacterial strain isolated from the gall-inducing insect (larval head) associated with gall induction in Manihot esculenta.tif]

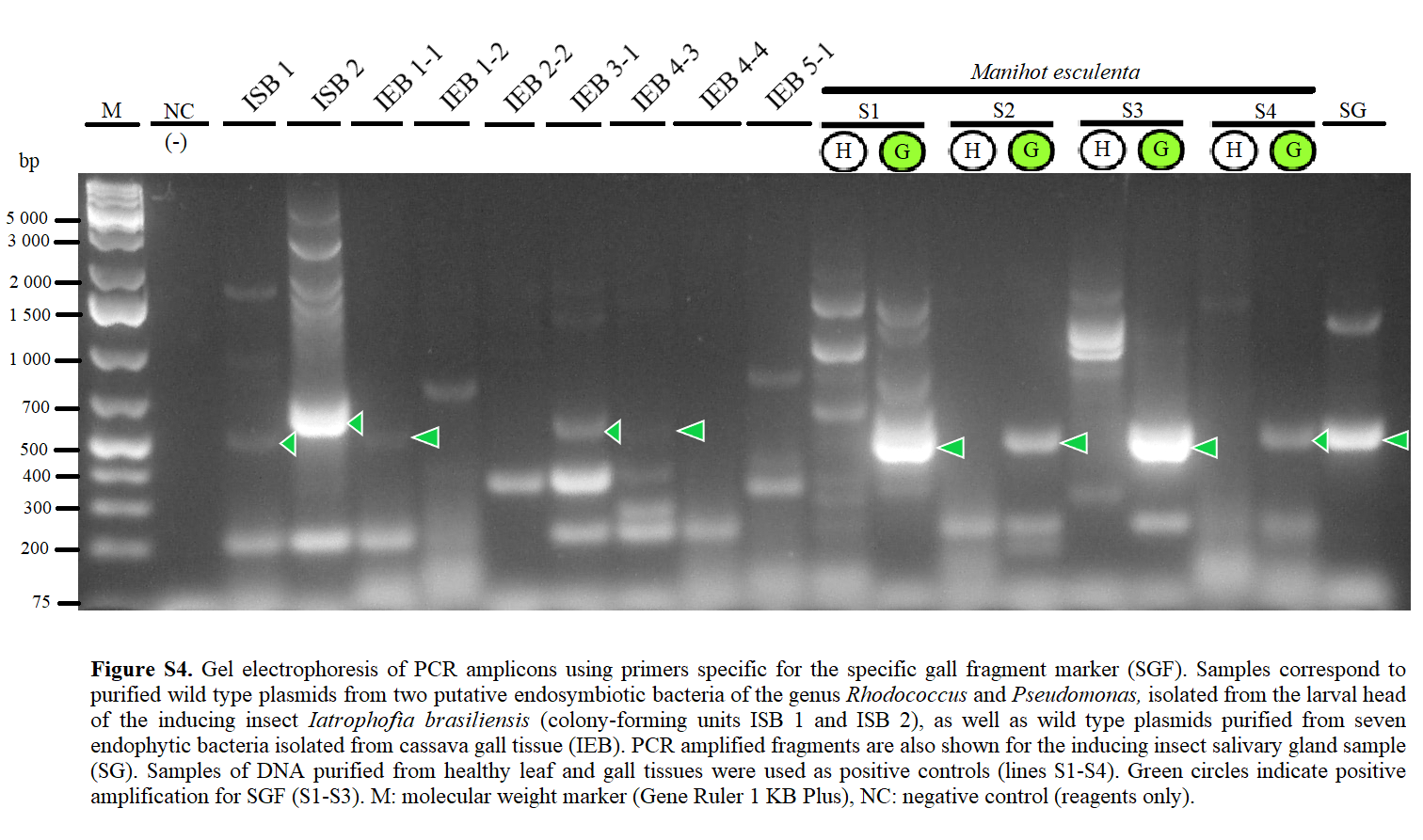

Supplement: Supplementary file 1 [file DataSheet_1.zip › Supplementary Material/Figure_S4_Gel electrophoresis of PCR amplicons using primers specific for the specific gall fragment marker (SGF).tif]

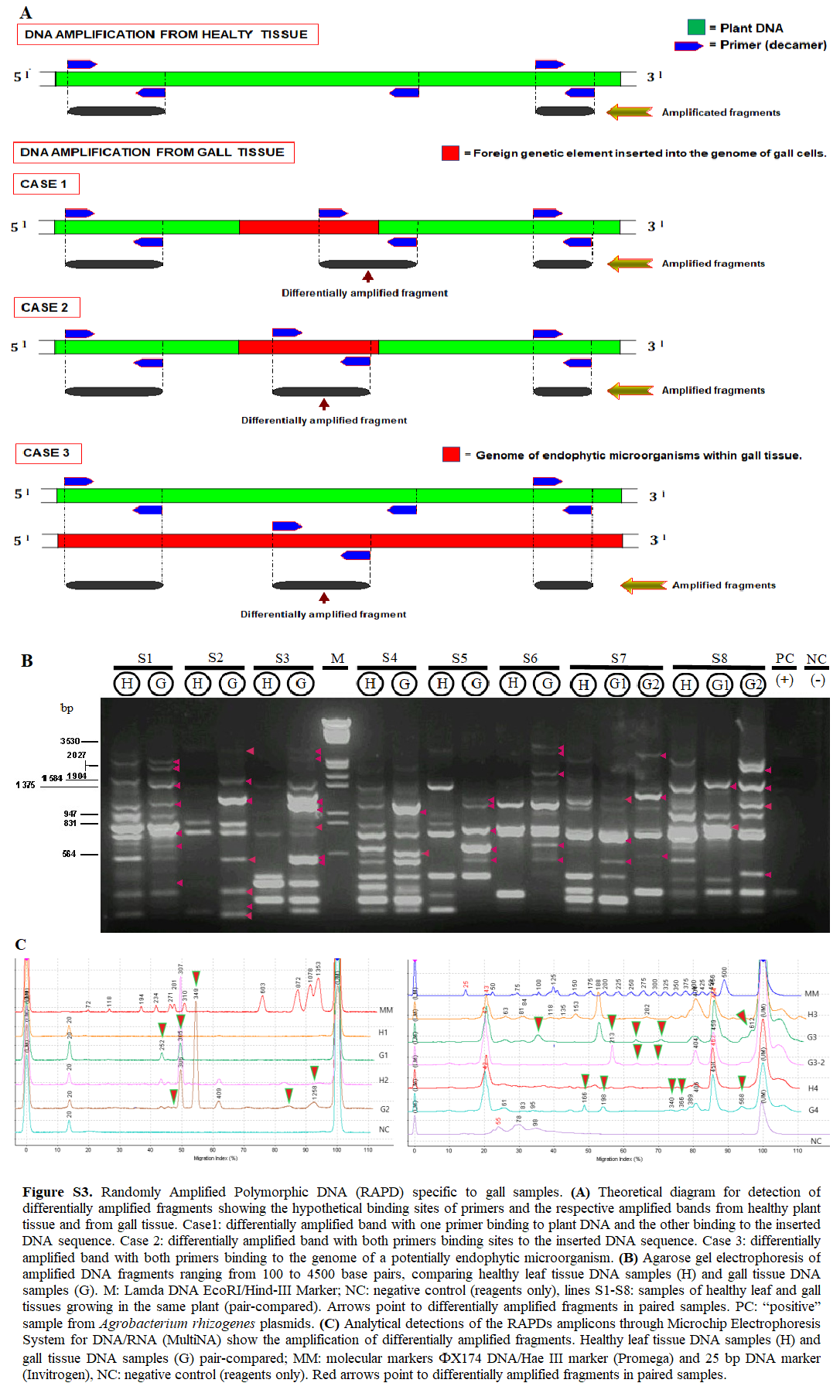

Supplement: Supplementary file 1 [file DataSheet_1.zip › Supplementary Material/Figure_S3_Randomly Amplified Polymorphic DNA (RAPD) specific to gall samples.tif]

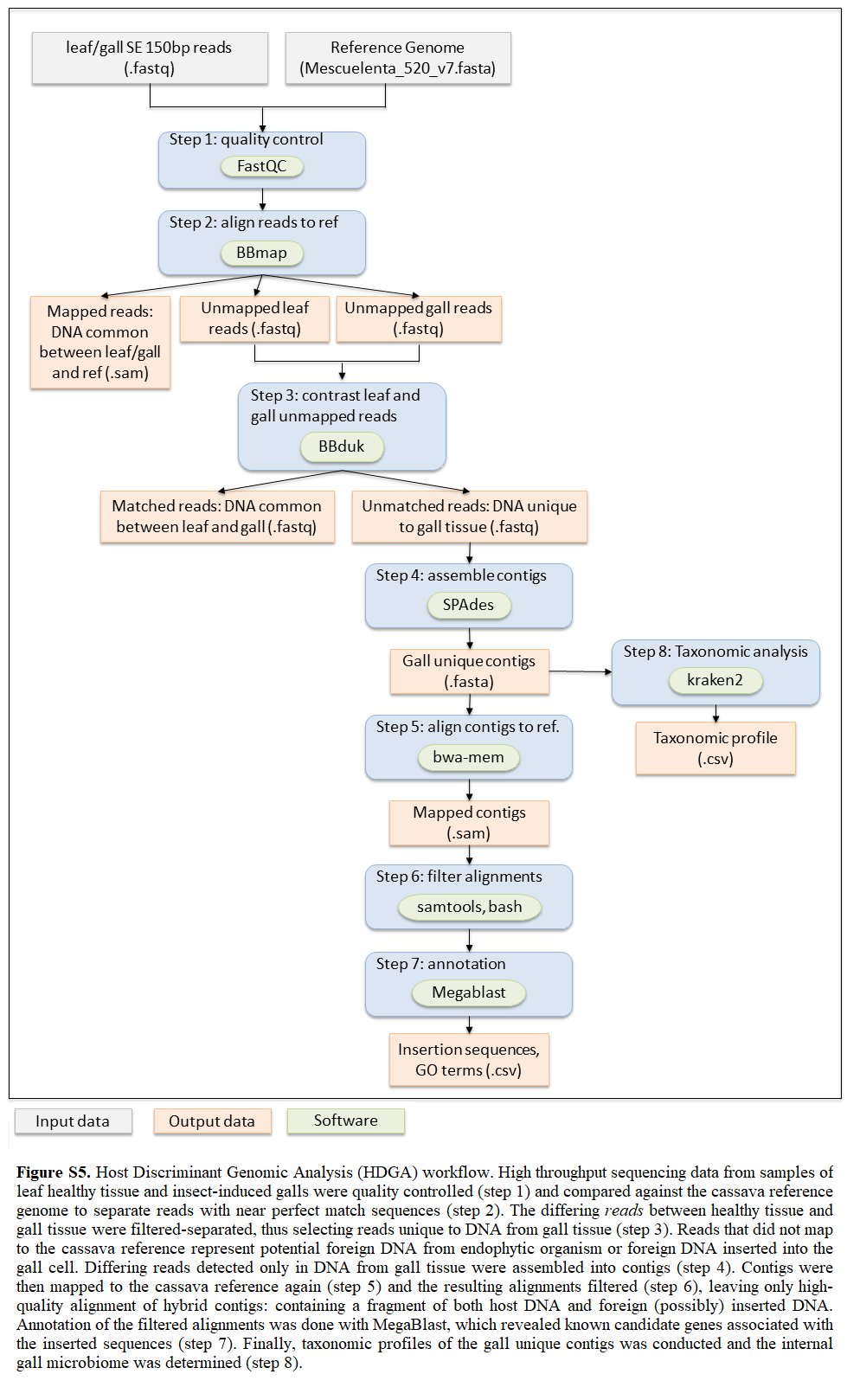

Supplement: Supplementary file 1 [file DataSheet_1.zip › Supplementary Material/Figure_S5_Host Discriminant Genomic Analysis (HDGA) workflow.tif]

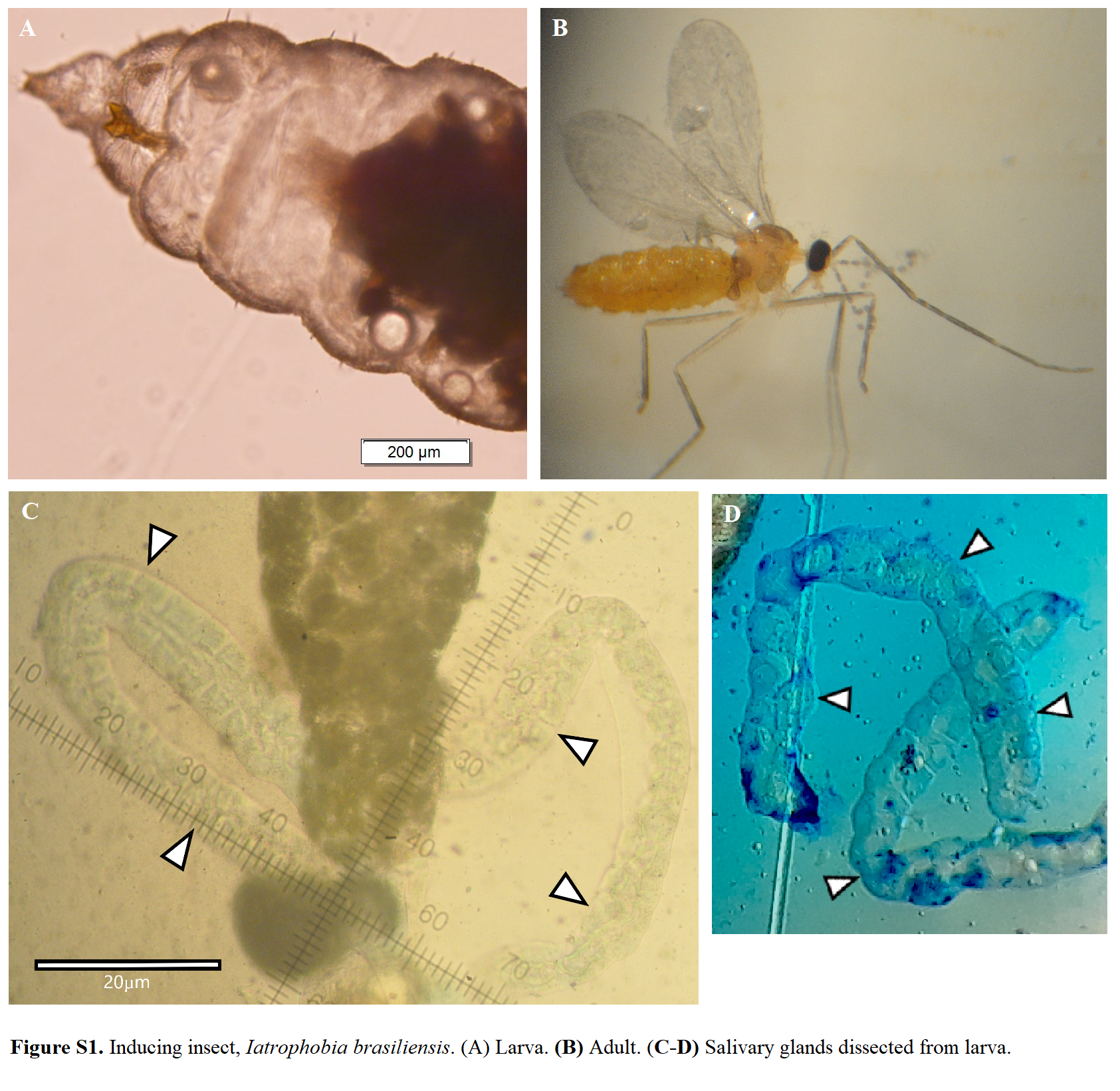

Supplement: Supplementary file 1 [file DataSheet_1.zip › Supplementary Material/Figure_S1_Inducing insect Iatrophobia brasiliensis.tif]
